# Deficiency of metabolic regulator PKM2 activates the pentose phosphate pathway and generates TCF1+ progenitor CD8+ T cells to improve checkpoint blockade

**DOI:** 10.21203/rs.3.rs-3356477/v1

**Published:** 2023-09-21

**Authors:** Geoffrey J. Markowitz, Yi Ban, Diamile A. Tavarez, Liron Yoffe, Enrique Podaza, Yongfeng He, Mitchell T. Martin, Michael J. P. Crowley, Tito A. Sandoval, Dingcheng Gao, M. Laura Martin, Olivier Elemento, Juan R. Cubillos-Ruiz, Timothy E. McGraw, Nasser K. Altorki, Vivek Mittal

**Affiliations:** Weill Cornell Medicine; Weill Cornell Medicine; Weill Cornell Medicine; Weill Cornell Medicine; Weill Cornell Medicine; Weill Cornell Medicine; Weill Cornell Medicine; Weill Cornell Medicine; Weill Cornell Medicine; Weill Cornell Medicine; Weill Cornell Medicine; Weill Cornell Medicine; Weill Cornell Medicine; Weill Cornell Medicine; Weill Cornell Medicine; Weill Cornell Medicine

**Keywords:** Non-small cell lung cancer, TCF1, CD8+ T cell, PKM2, metabolic reprograming, pentose phosphate pathway, Immunotherapy

## Abstract

TCF1^high^ progenitor CD8+ T cells mediate the efficacy of PD-1 blockade, however the mechanisms that govern their generation and maintenance are poorly understood. Here, we show that targeting glycolysis through deletion of pyruvate kinase muscle 2 (PKM2) results in elevated pentose phosphate pathway (PPP) activity, leading to enrichment of a TCF1^high^ central memory-like phenotype and increased responsiveness to PD-1 blockade *in vivo*. PKM2^KO^ CD8+ T cells showed reduced glycolytic flux, accumulation of glycolytic intermediates and PPP metabolites, and increased PPP cycling as determined by 1,2 ^13^C glucose carbon tracing. Small molecule agonism of the PPP without acute glycolytic impairment skewed CD8+ T cells towards a TCF1^high^ population, generated a unique transcriptional landscape, enhanced tumor control in mice in combination with PD-1 blockade, and promoted tumor killing in patient-derived tumor organoids. Our study demonstrates a new metabolic reprogramming that contributes to a progenitor-like T cell state amenable to checkpoint blockade.

## Introduction

CD8+ T cells are critical determinants of the anti-tumor immune response to immune checkpoint blockade^1–4^. These therapies target inhibitory receptors expressed on the CD8+ T cells including PD-1, which is upregulated upon activation and expressed as cells accumulate dysfunctional characteristics^5,6^. Recent studies have demonstrated that progenitor-exhausted CD8+ T cells expressing transcription factor TCF1 (T cell factor 1, encoded by *Tcf7*), are key mediators of anti-PD-1 therapy to inhibit tumor progression^7,8^.

While TCF1 has been associated with progenitor-exhausted T cell populations, it is also a critical regulator of memory CD8+ T cell development^9–11^. TCF1 expressing CD8+ T cells mediate more durable anti-tumor immunity compared to their TCF1^−^ effector-like counterparts^7,8,11,12^. Upon antigenic stimulation, TCR ligation drives differentiation of CD8+ T cells associated with marked alterations in the transcriptional and metabolic landscape^13–16^, with effector cells upregulating aerobic glycolysis to support their proliferative and cytotoxic phenotypes. Recent studies have begun to assess the potential of manipulating metabolic pathways to alter differentiation status of CD8+ T cells^17–19^, but it remains unclear how these transcriptional and metabolic changes affect T cell differentiation to mediate anti-tumor efficacy. In this study, we interrogated the metabolic landscape of tumor-infiltrating CD8+ T cells and show that loss of metabolic regulator PKM2 serves as a key hub controlling TCF1+ central memory-like progenitor cells to improve responsiveness to PD-1 checkpoint blockade. Our results demonstrate that while glycolysis is critical for effector cell activity, a temporary dampening may serve to skew T cell differentiation to provide durable anti-tumor immunity and checkpoint blockade synergy.

## Results

### A screen of glycolytic genes in intratumoral CD8 + T cells identifies Pyruvate Kinase M (PKM) as a potential regulator of cell fate.

**Figure 1:**
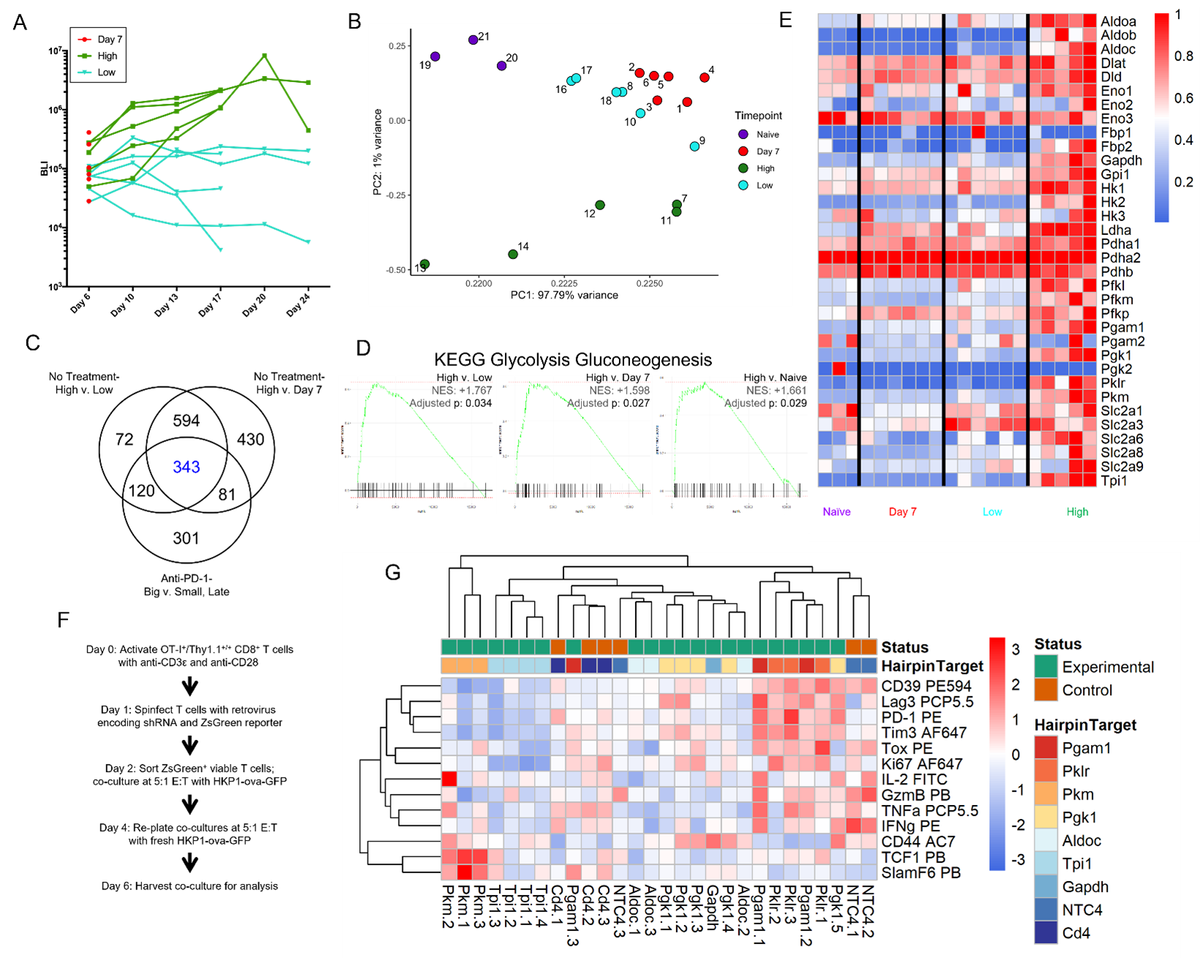
A genetic screen targeting glycolytic enzymes identifies Pyruvate kinase, muscle (PKM) as a potential regulator of T cell differentiation. **a,** Bioluminescent imaging (BLI) plots of tumor growth kinetics in mice from which CD8+ T cells were isolated at different time points (Day 7 for Day 7 group, Days 17 and 24 for Low and High groups) for bulk RNA sequencing analysis. Cohorts of mice (Naïve, Day 7, Low, and High) are depicted **b**, Principal component analyses of sequencing data from (a). **c**, Venn diagram showing overlaps of differentially expressed genes (p<0.05, adjusted p<0.2, absolute log_2_ fold change ≥ 1) between tumor phenotypes from this dataset and from comparisons between big and small tumors at later timepoints in the previously published anti-PD-1 dataset. **d**, Gene set enrichment analysis using the KEGG Glycolysis/Gluconeogenesis dataset for differences in glycolytic gene signatures in tumor-infiltrating CD8+ T cells between the High tumor burden group and others. **e**, Heatmap showing normalized expression of selected genes from the KEGG Glycolysis / Gluconeogenesis dataset across groups. **f**, Schematic of an shRNA screen targeting differentially expressed glycolytic enzymes from (e). **g**, Heatmap of normalized protein expression of cytokines, effector proteins, transcription factors, and surface markers associated with T cell differentiation and effector function measured by mean fluorescence intensity (MFI). **Numbers:** (a-e) Naïve group n=3 biological replicates, Day 7 group n=6 biological replicates, Low group n=6 biological replicates, High group n=5 biological replicates. (g) Data from 3–5 hairpins for each target were collected, except Gapdh, where two of the hairpins used proved to be lethal to the T cells. **Statistics:** (b) PCA calculated using princomp in R and plotted with ggplot2; (c) DEGs calculated using the limma package in R; (d) GSEA by fgsea package in R. **Abbreviations:** PE594, PE-Dazzle 594; PCP5.5, Peridinin Chlorophyll-A Protein-Cyanine5.5; PE, Phycoerythrin; AF647, Alexa Fluor 647; FITC, Fluorescein isothiocyanate; PB, Pacific Blue; AC7, Allophycocyanin Cyanine7.

To investigate the evolving transcriptome as a function of tumor progression, we used RNA sequencing analysis of tumor-infiltrating CD8 + T cells from the HKP1 orthotopic mouse model of NSCLC (Fig. 1A)^20^. Principal component analyses (PCA) showed clustering of the samples as a function of time and tumor burden with CD8 + T cells from large tumors exhibiting a distinct transcriptional landscape (Fig. 1B). We integrated this treatment-naïve dataset with our previously published anti-PD-1 treatment dataset to evaluate big (non-responding) vs small (responding) tumors^4^ to identify molecular targets for intervention, either as single agents or in combination with checkpoint blockade therapy. 343 differentially expressed genes were identified, including several regulators of metabolism (Fig. 1C, **Extended Data Table 1**). Gene set enrichment analysis (GSEA) identified enrichment of metabolic pathways^21^ in CD8 + T cells from large tumors compared with small or early-stage tumors or naïve lungs in both the treatment-naïve and anti-PD-1 treated samples, with glycolysis/gluconeogenesis being one of the top hits in all comparisons (Fig. 1D, **Extended Data** Fig. 1A, **Extended Data Table 2**). Differential gene expression showed increased expression of several glycolytic genes in CD8 + T cells from larger tumors (Fig. 1E), consistent with observations in tumors resistant to PD-1 treatment (group 3 and 6), indicating a metabolically active yet ineffective anti-tumor response (**Extended Data** Fig. 1B, **Extended Data Table 1**). Given that recent studies have intimately linked metabolism to T cell phenotypes and function^13–19,22^, we interrogated the impact of individual glycolytic genes on T cell differentiation and function by performing an shRNA screen using a co-culture system of tumor cells expressing Ova_257 – 264_, a well-defined antigen commonly used in lung cancer models^23,24^, and antigen-specific OT-I T cells^25^. As expected, Ova_257 – 264_-expressing GFP + HKP1 cells (HKP1-ova-GFP) presented Ova_257 – 264_ on MHC-I and upregulated PD-L1 expression upon stimulation with IFNγ (**Extended Data** Fig. 2A). OT-I cells were activated using either antigen-presenting cells with cognate peptide or plate-bound anti-CD3ε and anti-CD28 (Fig. 1F, Extended Data Fig. 2C), and subsequently co-cultured with HKP1-ova-GFP cells for up to 10 days after initial stimulation. T cells in co-culture initially expressed effector cytokines and granzyme B (GzmB), as well as increased TCF1 (**Extended Data** Fig. 2D-2F), along with modest expression of Tox and low levels of checkpoint proteins. However, over time, T cells acquired reduced cytokine and GzmB production, reduced TCF1 expression, and elevated expression of Tox and checkpoint proteins (**Extended Data** Fig. 2D-2F). Using this co-culture system, we performed an shRNA screen targeting individual glycolytic genes found to be enriched in our 343 differentially expressed genes (Fig. 1C). Consistent with the known requirement of glycolysis for optimal effector activity, knockdown of most glycolytic enzymes resulted in a hypofunctional effector state (Fig. 1G). Uniquely among the enzymes screened, inhibition of pyruvate kinase, muscle (PKM) resulted in upregulation of TCF1 and SlamF6, two markers of a more progenitor-like state (Fig. 1G)^6,8^. Taken together, these results demonstrate a unique effect of interference with pyruvate kinase activity to alter CD8 + T cell differentiation state.

### PKM2 loss impacts effector CD8 + T cell phenotypes in vitro.

**Figure 2:**
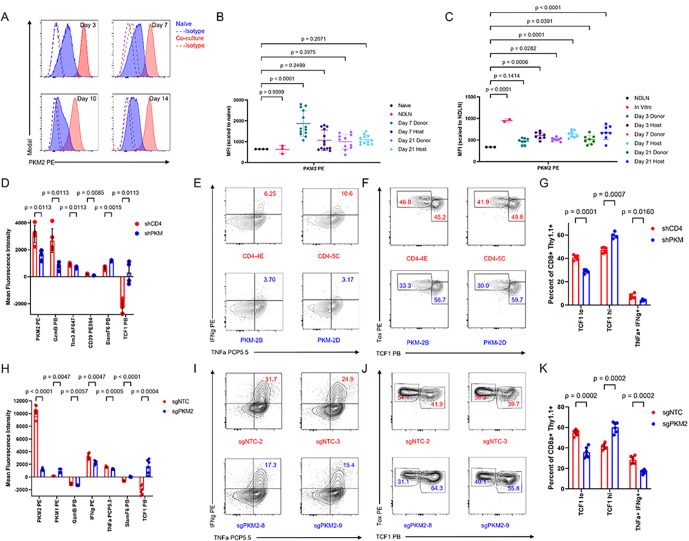
PKM2 is upregulated upon T cell activation *in vitro* and *in vivo*, and its deletion results in a less effector-differentiated phenotype. **a,** Histograms of fluorescence of PKM2 (solid lines) and isotype control (dotted lines) of naïve antigen-specific OT-I+ Thy1.1+ T cells (blue) or OT-I+ Thy1.1+ T cells from co-culture with Ova_257–264_–expressing HKP1-ova-GFP tumor cells (red) as a function of time (days 3–14 post-initial-stimulation). **b-c,** MFI of PKM2 expression from T cells isolated from HKP1-ova-GFP tumor-bearing C57Bl/6 mice. Naïve OT-I+ Thy1.1+ T cells were adoptively transferred into mice one day prior to orthotopic tumor implantation (b), while *in vitro* activated OT-I+ Thy1.1+ T cells were adoptively transferred 7 days post orthotopic tumor implantation into mice which received a single lymphodepleting dose of 5Gy of radiation (c). Sample fluorescence intensity was scaled to naïve OT-I+ Thy1.1+ T cell PKM2 expression acquired at each timepoint in (b), and from host T cells isolated from the non-draining lymph node (NDLN) in (c). **d-g,** Flow cytometry analysis of OT-I+ Thy1.1+ T cells co-cultured with HKP1-ova-GFP tumor cells as described (Fig. 1F). Activated T cells were infected with shRNAs targeting PKM (shPKM) or control CD4 (shCD4), and co-cultured with HKP1-ova-GFP tumor cells until 6 days post-initial-stimulation. **d,** MFIs for PKM2, effector molecules, surface markers and transcription factors in shCD4 (red) and shPKM (blue) T cells. **e,** Representative contour plots for IFNγ and TNFα staining in T cells infected with two hairpins targeting CD4 (red, CD4–4E and CD4–5C) and two hairpins targeting PKM (blue, PKM-2B and PKM-2D) at 6 days post-initial-stimulation. **f,** Representative contour plots for Tox and TCF1 staining in T cells infected with two hairpins targeting CD4 (red, CD4–4E and CD4–5C) and two hairpins targeting PKM (blue, PKM-2B and PKM-2D) at 6 days post-initial-stimulation. **g,** Quantification of populations of T cells infected with shCD4 (red) and shPKM (blue) at 6 days post-initial-stimulation. **h-k,** Flow cytometry analysis of OT-I+ Thy1.1+ T cells co-cultured with HKP1-ova-GFP tumor cells. Activated T cells were electroporated with guides targeting PKM2 (sgPKM2) or non-targeting controls (sgNTC), and co-cultured with tumor cells until 6 days post-initial-stimulation. **h,** MFIs for PKM isoforms, effector molecules, cytokines, surface markers, and transcription factors in sgNTC (red) and sgPKM2 (blue) T cells. **i,** Representative contour plots for IFNγ and TNFα staining in T cells electroporated with two non-targeting control guides (red, sgNTC-2 and sgNTC-3) and two guides targeting PKM2 (blue, sgPKM2–8 and sgPKM2–9) at 6 days post-initial-stimulation. **j,** Representative contour plots for Tox and TCF1 staining in T cells electroporated with two non-targeting control guides (red, sgNTC-2 and sgNTC-3) and two guides targeting PKM2 (blue, sgPKM2–8 and sgPKM2–9) at 6 days post-initial-stimulation. **k,** Quantification of populations of T cells electroporated with sgNTC (red) and sgPKM2 (blue) at 6 days post-initial-stimulation. **Numbers:** (b) n=3–13 biological replicates per group, aggregate of two experiments; (c) n=3–8 biological replicates per group; (d-g) n=2–3 biological replicates per hairpin, 4–5 per group, experiment repeated three times; (h-k) n=3 biological replicates per guide, 6 biological replicates per group, experiment repeated five times. **Statistcs:** (b, c) One-way ANOVA, Dunnet multiple comparisons; (d, g, h, k) Multiple unpaired t-tests, Holm-Šídák multiple comparisons. **Abbreviations:** PE, Phycoerythrin; PB, Pacific Blue; AF647, Alexa Fluor 647; PE594, PE-Dazzle 594; PCP5.5, Peridinin Chlorophyll-A Protein-Cyanine5.5.

PKM has two isoforms, PKM1 and PKM2, which differ in the exon in the 9th position: PKM1 transcript retains exon 9 and excises out exon 10, while PKM2 transcript excises out exon 9 and retains exon 10^26^. Analysis of the different isoform expression in tumor-infiltrating CD8 + T cells from the HKP1 orthotopic mouse model of NSCLC (Fig. 1A) demonstrated increased counts of PKM2 transcript compared with PKM1 (**Extended Data** Fig. 3A). In agreement with this sequencing data, co-culture assays of HKP1-ova-GFP tumor cells with OT-I T cells showed increased and sustained expression of PKM2, whereas PKM1 expression was not detected (Fig. 2A, **Extended Data** Fig. 3B). To assess PKM2 expression *in vivo*, we adoptively transferred naïve OT-I + Thy1.1 + CD8 + T cells into mice prior to implantation of HKP1-ova-GFP tumors and observed increased expression of PKM2 above naïve baseline (Fig. 2B). Similarly, adoptive transfer of *in vitro* activated OT-I + Thy1.1 + T cells into lymphodepleted mice 7 days after tumor implantation (Fig. 2C) also resulted in increased expression of PKM2 above baseline. These data demonstrate that PKM2 is upregulated upon activation and maintained throughout T cell differentiation.

Next, we used the co-culture system to determine the impact of PKM deficiency on T cell phenotype. We infected activated OT-I + T cells with shRNA targeting either PKM or a negative control, CD4 (**Extended Data** Fig. 3C-3D). Following co-culture with HKP1-ova-GFP cells, at 6 days after initial stimulation, shPKM T cells showed increased expression of SlamF6 and TCF1, whereas GzmB, Tim 3, and CD39 were reduced (Fig. 2D; **Extended Data** Fig. 4A-4B). Concurrently in these cultures, we observed reduced cytokine-producing populations with PKM knockdown and increased abundance of a TCF1 high population (Fig. 2E–2G). To specifically target the PKM2 isoform, we used CRISPR/Cas9 ribonucleoprotein complexes with isoform-specific guides to target exon 10 in activated OT-I + T cells; this resulted in robust loss of PKM2, and compensatory elevation of PKM1 expression (**Extended Data** Fig. 3E-3G). PKM2 knockout (PKM2^KO^) T cells following co-culture with HKP1-ova-GFP tumor cells showed higher levels SlamF6 and TCF1 with concomitant decreases in GzmB, IFNγ, and TNFa (Fig. 2H–K). As before, enhanced TCF1 levels were due to increased abundance of the TCF1 high population, while knockout resulted in reduced cytokine-producing populations (Fig. 2I–2K). PKM2 loss had a durable effect on altering differentiation, also displaying increased proportions of TCF1 high cells at day 9 post-initial-stimulation, when a more profound exhaustion phenotype^22^ had emerged in PKM2 wild-type (PKM2^WT^) T cells (**Extended Data** Fig. 5). Taken together, these data suggest that loss of PKM2 after T cell activation reduces effector differentiation.

### PKM2 loss alters CD8 + T cell differentiation states in NSCLC and melanoma models in vivo.

**Figure 3:**
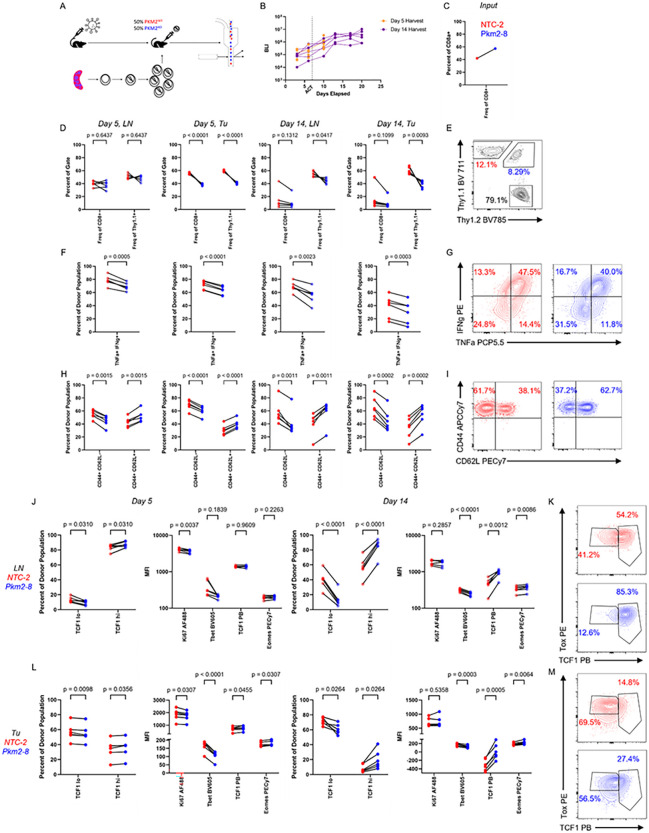
Loss of PKM2 results in a central memory-like phenotype in CD8+ T cells in NSCLC. Adoptive co-transfers of activated OT-I+ Thy1.1+ PKM2^WT^ (NTC-2, red) and PKM2^KO^ (Pkm2–8, blue) CD8+ T cells distinguished by Thy1.1 zygosity were performed into lymphodepleted C57Bl/6 mice 7 days after implantation of HKP1-ova-GFP tumors. Tumor burden was measured by bioluminescence imaging, and mice harvested and T cells subsequently phenotyped 5 and 14 days later. **a,** Experimental schematic. **b,** Bioluminescence imaging to measure tumor burden in mice harvested at day 5 (orange lines) or day 14 (purple lines) post adoptive co-transfer. **c,** Input proportions of PKM2^WT^ (NTC-2, red) and PKM2^KO^ (Pkm2–8, blue) CD8+ T cells transferred into mice. **d-i,** Quantification of populations of PKM2^WT^ (red) and PKM2^KO^ (blue) CD8+ T cells isolated from draining lymph nodes (LN) and tumors (Tu) 5 and 14 days after adoptive co-transfers. Frequencies of PKM2^WT^ (red) and PKM2^KO^ (blue) cells as percentages of total CD8+ T cells and donor Thy1.1+ cells (d), TNFα+ IFNγ+ proportions of PKM2^WT^ (red) and PKM2^KO^ (blue) cells after restimulation (f), and CD44+ CD62L- and CD44+ CD62L+ proportions (h) of PKM2^WT^ (red) and PKM2^KO^ (blue) were quantified. Representative contour plots from day 14 tumor samples are shown for each analysis (e, g, i), with PKM2^WT^ in red and PKM2^KO^ in blue. Panel (e) additionally has host population percentage in black. **j-m,** Quantification of populations of cells with differential TCF1 expression and quantification of transcription factor mean fluorescence intensities (MFIs) from PKM2^WT^ (red) and PKM2^KO^ (blue) CD8+ T cells isolated from draining lymph nodes (j) and tumors (l) 5 and 14 days after adoptive co-transfers. Representative contour plots for Tox and TCF1 staining from day 14 draining lymph nodes (k) and day 14 tumor samples (m) are shown with PKM2^WT^ in red and PKM2^KO^ in blue. **Numbers:** (d-m) n=6 biological replicates per group, experiment repeated three times. **Statistics:** (d-m) Multiple paired t-tests, Holm-Šídák multiple comparisons. **Abbreviations:** BV711, Brilliant Violet 711; BV785, Brilliant Violet 785; PE, Phycoerythrin; PCP5.5, Peridinin Chlorophyll-A Protein-Cyanine5.5; APCCy7, Allophycocyanin-Cyanine7; PECy7, Phycoerythrin-Cyanine7; AF488, Alexa Fluor 488; BV605, Brilliant Violet 605; PB, Pacific Blue.

We next determined the consequence of PKM2 deficiency on CD8 + T cell phenotypes in the tumor microenvironment (TME) and their draining lymph nodes (dLNs) *in vivo*. To control for possible phenotypic variation due to inter-mouse heterogeneity, we performed adoptive co-transfers of a mix of PKM2^KO^and PKM2^WT^ OT-I + T cells which could be distinguished *in vivo* by the zygosity in Thy1.1 expression (homozygous or heterozygous) into lymphodepleted HKP1-ova-GFP mice (Fig. 3A–3B). We phenotypically examined donor populations present in the tumors and dLNs at early and later timepoints after adoptive co-transfers (**Extended Data** Fig. 4C-4F). In the tumors, PKM2^WT^ cells rapidly became the dominant transferred population, whereas in the dLNs, PKM2^WT^ cells only significantly outnumbered PKM2^KO^ cells later in the experiment (Fig. 3C–3E). Similar to the phenotypes observed *in vitro* (Fig. 2D–2K), PKM2^KO^ cells upon re-stimulation showed reduced expression of IFNγ and TNFa in both tumors and dLNs compared to PKM2^WT^ cells (Fig. 3F–3G), indicative of decreased effector phenotype. Concordantly, PKM2^KO^ cells were enriched for CD44 + CD62L + cells in both tumors and dLNs, indicative of a central memory-like phenotype (Fig. 3H–3I). Further characterization of this altered differentiation state showed increased expression of the transcription factors TCF1 and Eomes and decreased Tbet expression in PKM2^KO^ cells in tumors and dLNs (Fig. 3J–3M).

To determine if the effects of PKM2 deletion on T cell phenotype were not limited to a Kras^G12D/+^ p53^−/−^ model of NSCLC, we employed the B16F10 melanoma model expressing Ova_257 – 264_ and GFP, B16F10-ova-GFP. We validated presentation of Ova_257 – 264_ on MHC-I and upregulated PD-L1 expression in this model upon stimulation with IFNγ (**Extended Data** Fig. 2B). Consistent with the NSCLC model, activation both *in vitro* and *in vivo* resulted in increased expression of PKM2, which subsided over time (**Extended Data** Fig. 6A-6B). Adoptive co-transfers of PKM2^KO^ and PKM2^WT^ OT-I + T cells into lymphodepleted B16F10-ova-GFP mice (**Extended Data** Fig. 6C-6D) showed dominance of PKM2^WT^ T cells in both the tumor and dLN tissue, with higher cytokine expression in PKM2^WT^ cells (**Extended Data** Fig. 6E-6I). As in NSCLC, PKM2^KO^ T cells exhibited a central memory-like phenotype, with elevated CD44 + CD62L + proportions at both timepoints in both tissues and enhanced TCF1 and Eomes expression at the later timepoint, marginally in the draining lymph node and significantly in the tumor (**Extended Data** Fig. 6J-6O). Together, the data demonstrate that PKM2 is expressed in tumor-specific T cells upon activation, and its loss results in a central memory-like state across multiple tumor types but with potential cancer-specific differences in magnitude of effect.

### In vivo PKM2 loss synergizes with checkpoint blockade to promote anti-tumor activity.

**Figure 4:**
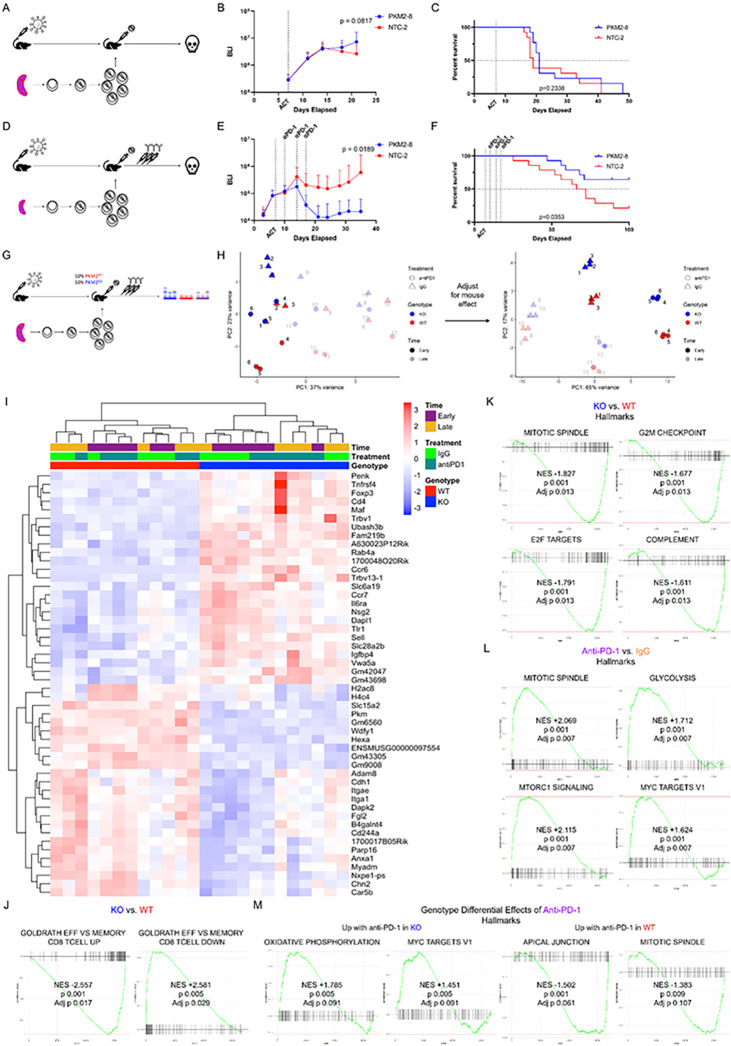
PKM2 deletion generates T cells with memory signatures which enhance the efficacy of PD-1 checkpoint blockade. **a-c,** Activated OT-I+ Thy1.1+ PKM2^WT^ (NTC-2, red) or PKM2^KO^ (Pkm2–8, blue) CD8+ T cells were adoptively transferred into lymphodepleted C57Bl/6 mice 7 days after orthotopic implantation of HKP1-ova-GFP tumors. **a,** Experimental schematic. **b,** Bioluminescence imaging to measure tumor burden. **c,** Overall mouse survival monitoring. **d-f** Activated OT-I+ Thy1.1+ PKM2^WT^ (NTC-2, red) or PKM2^KO^ (PKM2–8, blue) CD8+ T cells were adoptively transferred into lymphodepleted C57Bl/6 mice 7 days after orthotopic implantation of HKP1-ova-GFP tumors. 3 doses of anti-PD-1 were administered on days 10, 14, and 17 after tumor implantation. **d,** Experimental schematic. **e,** Bioluminescence imaging to measure tumor burden. **f,** Overall mouse survival monitoring. **g-m,** Transcriptomic analysis of adoptively-transferred T cells. Adoptive co-transfers of activated OT-I+ Thy1.1+ PKM2^WT^ (NTC-2, red) or PKM2^KO^ (PKM2–8, blue) CD8+ T cells distinguished by Thy1.1 zygosity were performed into lymphodepleted C57Bl/6 mice 7 days after implantation of HKP1-ova-GFP tumors. 3 doses of either IgG control or anti-PD-1 were administered on days 10, 14, and 17 after orthotopic implantation. T cells were subsequently sorted back from tumors based on Thy1.1, phenotyped by flow cytometry, and underwent bulk RNA sequencing. **g,** Experimental schematic. **h,** Principal component analysis of bulk RNA sequencing data before (left) and after (right) removal of mouse effect. **i,** Heatmap showing normalized expression of the top 25 up- and downregulated genes based on donor T cell genotype. **j-m,** Gene set enrichment analyses examining: signatures for effector and memory cells in PKM2^KO^ compared with PKM2^WT^ (j); hallmark signatures in PKM2^KO^ compared with PKM2^WT^ (k); hallmark signatures in anti-PD-1 treated mice compared with IgG (l); and hallmark signatures for differential effects of anti-PD-1 based on donor T cell genotype (m). **Numbers:** (b-c) n=13 biological replicates per group, experiment repeated twice; (e-f) n=14 biological replicates per group, experiment repeated twice. (h-m) n=3 biological replicates per group. **Statistics:** (b) Two-way ANOVA, Šídák’s multiple comparisons with Grubbs’ outlier test with alpha = 0.0001; (c) Log-rank Mantel-Cox test; (e) Two-way ANOVA, Šídák’s multiple comparisons with Grubbs’ outlier test with alpha = 0.0001; (f) Log-rank Mantel-Cox test; (h) PCAs generated with plotPCA in the DESeq2 package and graphed with ggplot2 in R; mouse effect removed with the removeBatchEffect method in the limma package in R; (i) DEGs calculated by using the DESeq2 package in R; (j-m) GSEA by fgsea package in R.

We observed that PKM2 loss in tumor-specific CD8 + T cells altered their differentiation state, resulting in a TCF1^high^ central memory-like phenotype. However, the ensuing CD8 + T cell phenotypes were not sufficient to impair tumor growth or improve overall survival (Fig. 4A–4C). Importantly, intratumoral TCF1^+^ CD8 + T cells have been shown to mediate the efficacy of PD-1 checkpoint blockade^7,8,12^. To determine if the TCF1^high^ CD8 + T cells resulting from PKM2 deficiency respond to PD-1 inhibition to exert anti-tumor effects, we tested a combination of anti-PD-1 and PKM2 loss in CD8 + T cells in the HKP1-ova-GFP mouse model. PKM2^WT^ or PKM2^KO^ OT-I + T cells were adoptively transferred into cohorts of HKP1-ova-GFP mice, and subsequently administered 3 doses of anti-PD-1 antibody as described before^4,27^. PKM2^KO^ CD8 + T cells in combination with PD-1 blockade significantly impaired tumor growth and improved survival (Fig. 4D–4F). Adoptive transfers of single genotype T cell populations into tumor-bearing mice with subsequent treatment with either anti-PD-1 or IgG revealed significant tumor control as early as 4 days after the first dose of anti-PD-1 (**Extended Data** Fig. 7A). PKM2^WT^ and PKM2^KO^ T cells demonstrated similar abundance in lymph nodes and tumors, with TCF1^high^ cells dominating in draining lymph nodes, and enrichment of TCF1^low^ cells upon anti-PD-1 treatment, but with large inter-tumor variability (**Extended Data** Fig. 7B-7C).

To further investigate the effects of PD-1 inhibition, we adoptively co-transferred PKM2^WT^ and PKM2^KO^ T cells into tumor-bearing mice, followed by administration of anti-PD-1 or IgG, and performed comprehensive flow cytometric phenotyping of donor populations. We again observed rapid tumor control by anti-PD-1 treatment (**Extended Data** Fig. 7D). Furthermore, T cell phenotypes from anti-PD-1 treated mice largely mirrored their IgG or non-treated counterparts (Fig. 3). In comparison to PKM2^WT^ T cells, we observed 1) decreased proportions of PKM2^KO^ T cells (**Extended Data** Fig. 7E-7F), 2) increased proportions of memory-like cells (CD44 + CD62L+, CD127 + Klrg1−) and reduced proportions of effector-like cells (CD44 + CD62L−, CD127-Klrg1+) with PKM2^KO^ (**Extended Data** Fig. 7G-7J), 3) increased proportions of TCF1^high^ cells with PKM2^KO^ (**Extended Data** Fig. 7K-7L), and 4) reduced IFNγ + TNFa + proportions with PKM2^KO^ (**Extended Data** Fig. 7M-7N). In both PKM2^WT^ and PKM2^KO^ T cells, TCF1^high^ cells displayed increased Ki67 expression compared with their TCF1^low^ counterpart, suggesting that TCF1^high^ cells were more proliferative Fig. 7O-7P). Anti-PD-1 treatment resulted in similar phenotypes in both genotypes of T cells: increased proportions of CD44 + CD62L− cells in tumors at later timepoints, a more rapid progression to the TCF1^low^ phenotype in the tumor, and a trend for more Ki67 expression in TCF1^high^ cells in the tumor at the latest timepoint. Importantly, we observed maintained higher proportions of memory-like T cells in PKM2^KO^ even with anti-PD-1 treatment, a population critical to the checkpoint blockade response^7,8^. These data suggest that the anti-PD-1 response machinery and mechanism is largely retained with PKM2 loss, with a sustained memory skewing in the presence of checkpoint blockade.

To obtain further insights into how PD-1 inhibition impacts PKM2^KO^ CD8 + T cells, we performed RNA sequencing (RNA-seq) analyses of sorted tumor-infiltrating T cells from HKP1-ova-GFP tumor-bearing mice adoptively co-transferred with a mix of PKM2^KO^ and PKM2^WT^ OT-I + T cells and treated with IgG control or anti-PD-1 (Fig. 4G, **Extended Data Fig. 8A-8C**). As expected, PKM2^WT^ cells increased in numbers and PKM2^KO^ cells lacking PKM2 expression were associated with increased proportions of CD44 + CD62L + cells (**Extended Data Fig. 8D-8E**). When normalized for inter-mouse heterogeneity, samples clustered based on genotype, treatment status, and time (Fig. 4H). Differential gene expression and gene set enrichment analysis (GSEA) showed enrichment of a memory signature^28^ in PKM2^KO^ cells and enrichment of an effector signature in PKM2^WT^ cells, consistent with flow cytometry-based phenotyping (Fig. 4I–4J, **Extended Data Table 3**). These differential enrichments were present at both the early and later timepoint, demonstrating a persistent skewing towards a memory-like phenotype with PKM2 loss (**Extended Data Fig. 8F**). Further interrogation of transcriptome data using hallmark datasets^29^ displayed a more proliferative phenotype in PKM2^WT^ cells, consistent with their elevated abundance compared with PKM2^KO^ cells, as well as an enhanced complement signature (Fig. 4K). The differential signature enrichment by genotype shifted over time: at early timepoints, PKM2^WT^ cells displayed enrichment of signatures associated with cell proliferation and glycolysis (**Extended Data Fig. 8G, Extended Data Table 4**), while at later timepoints these cells had more inflammation-associated signatures (**Extended Data Fig. 8H, Extended Data Table 4**). Anti-PD-1 treatment meanwhile enriched for a proliferative and metabolically active state compared with IgG (Fig. 4L); these trends also shifted over time, reflecting similar patterns to the differences observed between PKM2^WT^ and PKM2^KO^ cells, with early differences in proliferation-associated signatures and later differences in inflammation-associated signatures (**Extended Data Fig. 8I-8J**, **Extended Data Tables 5–6**). Finally, to interrogate how PKM2^WT^ and PKM2^KO^ cells responded differently to anti-PD-1 treatment, we performed gene set enrichment analysis (GSEA) to identify pathways which were differentially affected based on genotype upon anti-PD-1 treatment (Fig. 4M, **Extended Data Table 7**). A subset of the response was conserved between genotypes, including activation of the hallmark signatures E2F targets, G2M checkpoint, and glycolysis, among others There were also divergently enriched signatures, suggesting a qualitative difference to these cellular populations. In PKM2^WT^ cells, anti-PD-1 treatment induced enrichment of hallmark pathways associated with proliferation, including apical junction and mitotic spindle, while PKM2^KO^ cells showed enrichment of oxidative phosphorylation (OXPHOS), suggesting a metabolic shift upon anti-PD-1 therapy (Fig. 4M, **Extended Data Table 7**) This was further supported by gene ontology: biological process (GO:BP) datasets, where PKM2^KO^ cells showed enrichment for terms such as ATP synthesis coupled electron transport, mitochondrial respiratory chain complex assembly, OXPHOS, and electron transport chain, along with other similar terms (**Extended Data Table 7**). Intriguingly, both OXPHOS and fatty acid oxidation are associated with memory cell fuel utilization, supporting a memory cell phenotype for PKM2^KO^ cells and suggesting an active metabolic response to anti-PD-1 therapy^14,18,19,30^. Taken together, these data confirm the altered differentiation status induced by PKM2 loss, with robust effects on proliferation and inflammatory processes, and demonstrate the impact of this altered state on response to checkpoint blockade.

### PKM2 loss results in reduced glycolytic flux, accumulation of glycolytic intermediates and activation of the pentose phosphate pathway.

**Figure 5:**
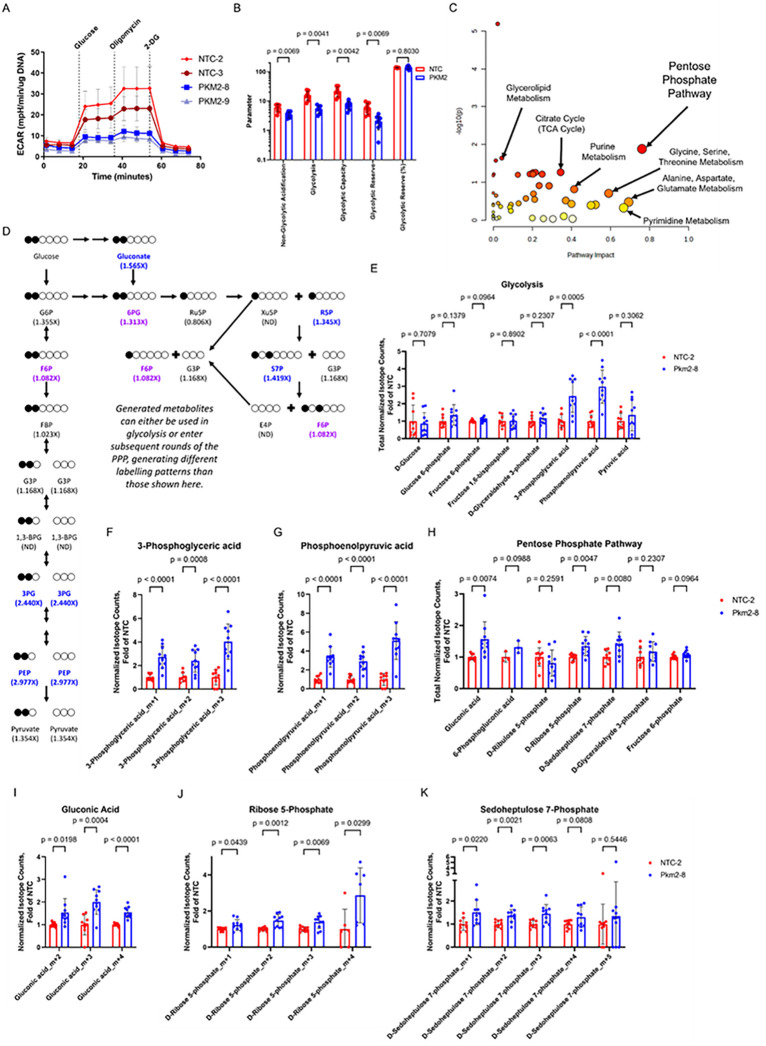
PKM2 deletion in T cells results in decreased glycolytic flux and increased pentose phosphate pathway activity. **a-b,** Activated OT-I+ Thy1.1+ CD8+ T cells were electroporated with guides targeting PKM2 (PKM2–8 and PKM2–9, blue) or non-targeting controls (NTC-2 and NTC-3, red), and co-cultured with HKP1-ova-GFP tumor cells. DAPI− CD8+ Thy1.1+ T cells were subsequently sorted from co-culture at day 6 post-initial-stimulation and underwent a glycolysis stress test by sequential treatment with glucose, oligomycin, and 2-deoxyglucose (2-DG). **a,** Extracellular acidification rate (ECAR) measured with a Seahorse bioanalyzer. **b,** Calculation of different glycolytic parameters from data in (a). **c-k,** Activated OT-I+ Thy1.1+ CD8+ T cells were electroporated with guides targeting PKM2 (PKM2–8, blue) or non-targeting control (NTC-2, red), and co-cultured with HKP1-ova-GFP tumor cells. On day 6 post-initial-stimulation, DAPI− CD8+ Thy1.1+ T cells were sorted and labelled with 1,2 ^13^C glucose for 2 hours, and panel of 204 polar metabolites profiled by liquid chromatography/mass spectrometry for abundance and labeling (LC/MS). Metabolite abundance was normalized by total isotope counts, with resultant data calculated as fold of average NTC-2 abundance for the given batch. **c,** Metaboanalyst identification of the pentose phosphate pathway as the most impacted pathway. **d,** Labeling pattern and quantification for glycolysis and pentose phosphate pathway metabolites after one round, with statistically significantly enriched (p<0.05) labelled metabolites shown in dark blue and labelled metabolites with p<0.1 in purple in (d). Multiple rounds of the pentose phosphate pathway can occur, with F6P isomerizing with G6P or integration of glycolysis-derived G3P, leading to different labeling patterns and isotopes. **e-k,** Quantification for all ^13^C labelled metabolites in glycolysis (e) and the pentose phosphate pathway (h), and different labelled isotopes of metabolites with significant enrichment in PKM2 knockout T cells over control: 3-phosphoglyceric acid (f), phosphoenolpyruvic acid (g), gluconic acid (i), ribose 5-phosphate (j), and sedoheptulose 7 phosphate (k). **Numbers:** (a-b) n=4 biological replicates per guide, experiment repeated three times; (c-k) n=9–10 biological replicates per group, aggregate of three experiments. **Statistics:** (b) Two-way ANOVA, Dunnett’s multiple comparisons; (e-k) Multiple unpaired t-tests. **Abbreviations:** G6P, glucose-6-phosphate; 6PG, 6-phosphogluconate; Ru5P, ribulose-5-phosphate; Xu5P, xylulose-5-phosphate; R5P, ribose-5-phosphate; F6P, fructose-6-phosphate; G3P, glyceraldehyde-3-phosphate; S7P, sedoheptulose-7-phosphate; FBP, fructose 1,6-bisphosphate; E4P, erythrose-4-phosphate; 1,3-BPG, 1,3-bisphosphglycerate; 3PG, 3-phosphoglycerate; PEP, phosphoenolpyruvate.

PKM2 is a rate-limiting enzyme in glycolysis, catalyzing the conversion of phosphoenolpyruvate (PEP) to pyruvate^26^. In addition to its metabolic role, PKM2 can serve as a transcriptional regulator, modify activity of several signaling cascades such as HIF1α, mTOR, STAT1, STAT3, STAT5, and TGFβ/Smad2/3^31–34^. Given these multiple roles, we examined the subcellular localization of PKM2 in activated CD8 + T cells isolated from co-cultures and observed robust cytoplasmic expression and little to no nuclear PKM2, suggesting an important metabolic role for PKM2 in CD8 + T cells (**Extended Data** Fig. 3G). To investigate the effects of PKM2 loss on T cell metabolism, we sorted T cells from co-cultures of HKP1-ova-GFP cells with either PKM2^WT^ or PKM2^KO^ OT-I + T cells at 6 days post-initial-stimulation and performed a glycolysis stress test using a Seahorse XF bioanalyzer. PKM2^KO^ T cells showed reduced glycolysis and glycolytic capacity compared with PKM2^WT^ counterparts, as expected (Fig. 5A–5B). This PKM2 deficiency resulting in reduced glycolysis did not impact fatty acid and glutamine oxidation (**Extended Data Fig. 9A-9D**). Therefore, to further characterize the evolving metabolic alterations resulting from PKM2 deficiency, we conducted steady-state polar metabolite profiling by liquid chromatography/mass spectrometry (LC/MS) at multiple timepoints following CRISPR/Cas9 RNP-mediated deletion of PKM2 (**Extended Data Fig. 9E-9L**, **Extended Data Table 8**). Compared with PKM2^WT^, PKM2^KO^ T cells sorted from co-cultures showed accumulation of various glycolytic intermediates over time (**Extended Data Fig. 9E-9H**). Both PEP, which has been shown to interfere with other glycolytic reactions^35^, and 3-phosphoglycerate, the substrate of phosphoglycerate mutase, an enzyme negatively regulated by PEP, demonstrated robust accumulation at early timepoints in PKM2^KO^ T cells in co-culture (**Extended Data Fig. 9F**). PKM2^KO^ T cells further displayed coincident enrichment of metabolites of the pentose phosphate pathway (PPP) with this accumulation of glycolytic intermediates (**Extended Data Fig. 9I-9L**). Glycolysis and the PPP share both glucose as a fuel source as well as several metabolites, including glucose 6-phosphate, fructose-6-phosphate, and glyceraldehyde 3-phosphate, which can be utilized by the PPP to generate NADPH and ribose-5-phosphate in activated T cells^16^. We observed consistent enrichment in sedoheptulose 7-phosphate in co-culture (**Extended Data Fig. 9I-9L**), with varying accumulation of other metabolites. Notably, fructose 6-phosphate, a product of another reaction negatively regulated by PEP, is also an important product of transketolase and transaldolase activity in the non-oxidative phase of the PPP; it was particularly enriched at later timepoints (**Extended Data Fig. 9G-9H**). These data demonstrate that PKM2 loss results in reduced glycolysis, and an increase in abundance of PPP metabolites.

To examine the effect of PKM2 loss on glucose processing more directly, we performed metabolic profiling using 1,2 ^13^C-labelled glucose^36,37^. Use of glucose labelled at positions 1 and 2 allows for discrimination of metabolites generated by glycolytic or PPP activity, while also allowing for observation of multiple rounds of PPP activity through differential labelling patterns^36,37^. We activated OT-I + T cells as before, then either deleted or retained PKM2 using electroporation of CRISPR/Cas9 RNPs, and subsequently co-cultured these T cells with HKP1-ova-GFP cells. At days 4 and 6 post-initial-stimulation, we sorted T cells from co-cultures, incubated them for 2 hours with 1,2 ^13^C-labelled glucose, then isolated metabolites and performed polar metabolite profiling by LC/MS (**Extended Data Table 9**). Metaboanalyst^38,39^ analyses of labelled metabolites demonstrated robust impact of PKM2 loss on the PPP at both timepoints (Fig. 5C, **Extended Data Fig. 9M**). At day 6 post-initial-stimulation, in PKM2^KO^ T cells we observed accumulation of labelled 3-phosphoglycerate and phosphoenolpyruvate, two metabolites upstream of PKM2, suggesting a blockade from the knockout (Fig. 5D–5G). We also observed increased proportions of labelled gluconate, ribose 5-phosphate, and sedoheptulose 7-phosphate (Fig. 5D, 5H–5K); gluconate is an entry point into the oxidative PPP, while ribose 5-phosphate and sedoheptulose 7-phosphate are two critical products generated in the non-oxidative PPP. Interestingly, we observed increased proportions of different order isotope charges in these glycolytic and PPP products than would be generated by a single round of glycolysis or the PPP, suggesting multiple PPP cycles in PKM2 knockout T cells (Fig. 5F–5G, 5I–5K). Day 4 post-initial-simulation data display similarities and some differences, one of which being a more significant impact on the TCA Cycle (**Extended Data Fig. 9M**). PKM2 loss resulted in significant differences in labelled glyceraldehyde 3-phosphate, 3-phosphoglycerate, phosphoenolpyruvate, ribose 5-phosphate, sedoheptulose 7-phosphate, and oxoglutarate, with increased normalized isotope counts observed in knockouts in each of these metabolites except for sedoheptulose 7-phosphate, which was decreased (**Extended Data Fig. 9N-9V**). Once again, labelling patterns show isotope charges indicating multiple rounds of PPP activity (**Extended Data Fig. 9Q-9V**). Taken together, these data substantiate our steady-state findings that PKM2 loss results in altered glycolytic flux and an increase in PPP metabolite generation, with multiple rounds of PPP activity.

### Pentose phosphate pathway agonism results in a TCF1 + state independent of glycolytic blockade and divergent from the phenotype induced by hexokinase inhibition.

**Figure 6:**
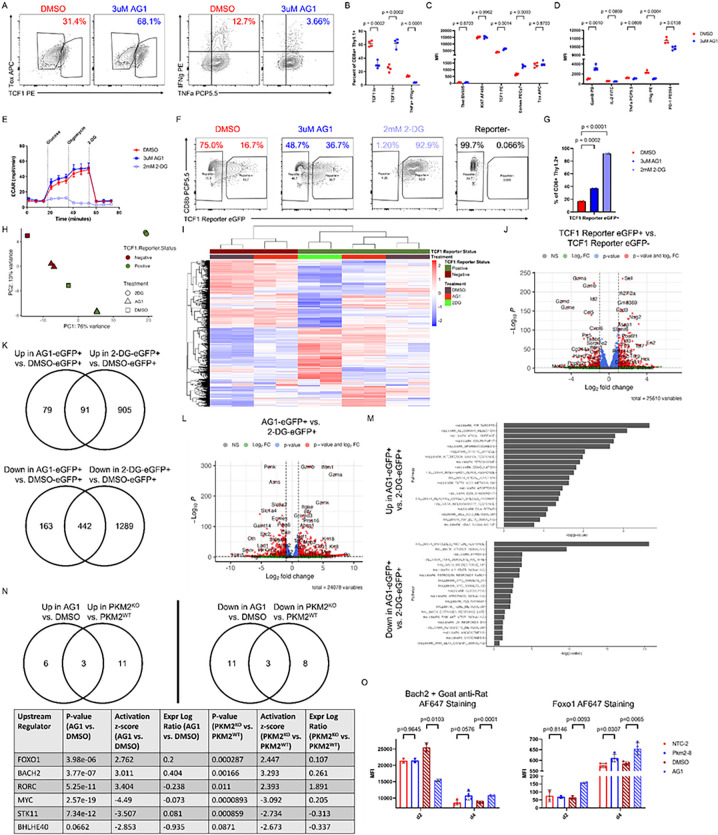
Pentose phosphate pathway agonism in T cells generates a memory-like phenotype distinct from that induced by blockade of glucose utilization. **a-d,** Flow cytometry analysis of activated OT-I+ Thy1.1+ T cells treated with either DMSO control (red) or 3μM glucose-6-phosphate dehydrogenase agonist AG1 (blue) and co-cultured with HKP1-ova-GFP tumor cells. T cells were activated for 24 hours, treated with DMSO or AG1 for another 24 hours, then co-cultured with HKP1-ova-GFP tumor cells at a 5:1 effector:target ratio until 6 days post-initial-stimulation with continuing DMSO or AG1 treatment. **a,** Representative contour plots for Tox and TCF1 staining in T cells treated with DMSO (left) or AG1 (right), with gates for populations with differential TCF1 expression. **b,** Quantification of populations from (a) as percent of cultured CD8+ Thy1.1+ T cells in DMSO (red) and AG1 (blue) treated co-cultures. **c,d,** Mean fluorescence intensities (MFIs) for transcription factors (c) and effector proteins, cytokines, and PD-1 (d) in co-cultured T cells treated with DMSO (red) or AG1 (blue). **e,** OT-I+ Thy1.1+ CD8+ T cells were activated for 48 hours, then co-cultured with HKP1-ova-GFP tumor cells. At 6 days post-initial-stimulation, DAPI− CD8+ Thy1.1+ T cells were sorted from co-culture, pre-treated for 2 hours with either DMSO (red), 3μM AG1 (blue) or 2mM hexokinase inhibitor 2-deoxyglucose (2-DG, periwinkle), then underwent a glycolysis stress test by sequential treatment with glucose, oligomycin, and 2-DG. Plotted is the extracellular acidification rate (ECAR) measured with a Seahorse bioanalyzer. **f-m,** Sequencing analysis of activated OT-I+ Tcf7^GFP^+ T cells treated with either DMSO control (red), 3μM AG1 (blue), or 2mM 2-DG (periwinkle) and co-cultured with HKP1-ova-GFP tumor cells. T cells were activated for 24 hours, treated with DMSO, AG1, or 2-DG for another 24 hours, then co-cultured with HKP1-ova-GFP tumor cells at a 5:1 effector:target ratio with continuing drug treatment. At 6 days post-initial-stimulation, DAPI− CD8b+ Thy1.2+ T cells were sorted on eGFP for TCF1 reporter expression, and subjected to RNA sequencing. **f,** Representative contour plots for CD8b and eGFP expression in T cells treated with DMSO (red), AG1 (blue), or 2-DG (periwinkle), with gates for populations with differential TCF1 eGFP reporter expression based on fluorescence in activated CD8+ T cells from an OT-I+ Tcf7^GFP^-strain cells (far right, black). **g,** Quantification of populations from (f) as percent of CD8+ Thy1.2+ T cells. **h,** Principal component analysis of bulk RNA sequencing data from sorted T cell populations from (f). Insufficient eGFP− cells were present to be included in the sequencing analysis. **i,** Heatmap showing normalized expression of the top 1000 variable genes based on TCF1 eGFP reporter status. **j,** Volcano plot showing differential gene expression (thresholds: absolute log_2_ Fold Change > 1, adj. p value <0.05) based on TCF1 eGFP reporter status, with genes up in eGFP+ cells on the right. **k,** Venn diagrams showing overlap of genes significantly differentially expressed (absolute Fold Change > 1.5, adj. p value <0.05) in the given conditions. **l,** Volcano plot showing differential gene expression (thresholds: absolute log_2_ Fold Change > 1, adj. p value <0.05) between eGFP+ samples generated by AG1 treatment or 2-DG treatment, with genes from AG1 treated samples on the right. **m,** Gene set enrichment analyses examining hallmark signatures in AG1-treated eGFP+ samples compared with 2-DG-treated eGFP+ samples. Pathways up in AG1-treated samples are on the top; pathways up in 2-DG-treated samples are on the bottom. **n,** IPA analysis for conserved upstream regulators between AG1 vs. DMSO treated T cells from *in vitro* co-culture from Fig. 6F–6N, and PKM2^KO^ vs. PKM2^WT^ T cells from *in vivo* adoptive co-transfers from Fig. 4G–4M; absolute activation z-score > 2. **o,** Flow cytometry analysis of activated OT-I+ Thy1.1+ T cells with PKM2 knockout or control, or AG1 treatment or vehicle and co-cultured with HKP1-ova-GFP tumor cells. T cells were activated for 24 hours, either electroporated with sgRNAs or treated with DMSO or AG1 and expanded for another 24 hours, then co-cultured with HKP1-ova-GFP tumor cells at a 5:1 effector:target ratio with continuing DMSO or AG1 treatment where relevant. Mean fluorescence intensities (MFIs) for Foxo1 and Bach2 were evaluated at day 2 and day 4 post initial stimulation. **Numbers:** (b-d) n=4 biological replicates per group, experiment repeated four times; (e-m) n=2 biological replicates per group; (n) n=4 biological replicates per group for AG1 vs. DMSO, n=12 replicates per group for PKM2^KO^ vs. PKM2^WT^; (o) n=2 biological replicates per group for day 2 and n=4 biological replicates per group for day 4, experiment repeated twice. **Statistics:** (b-d) Multiple unpaired t-tests, Holm-Šídák multiple comparisons; (g) One-way ANOVA, Dunnett’s multiple comparisons; (h) PCAs calculated using generated with plotPCA in the DESeq2 package in R (i,j,l) DEGs calculated using the DESeq2 package in R; (m) GSEA by fgsea package in R; (n) IPA Upstream Regulator Analysis, absolute Activation Score ≥ 2; (o) multiple unpaired t-tests, Holm-Šídák multiple comparisons. **Abbreviations:** APC, Allophycocyanin; PE, Phycoerythrin; PCP5.5, Peridinin Chlorophyll-A Protein-Cyanine5.5; BV605, Brilliant Violet 605; AF488, Alexa Fluor 488; PECy7, Phycoerythrin-Cyanine7; PB, Pacific Blue; FITC, Fluorescein isothiocyanate; PE594, PE-Dazzle 594; eGFP, enhanced Green Fluorescent Protein.

Our data demonstrated that PKM2 loss results in accumulation of PPP activity. We therefore asked if induction of this altered metabolic state was sufficient to phenocopy the altered differentiation observed upon PKM2 loss. Recent studies have interrogated effects of manipulation of the oxidative phase of the PPP on CD8 + T cell effector function, with conflicting outcomes^40–43^. To test the impact of elevated PPP activity upon T cell differentiation and function, we treated T cells with AG1, a small molecule agonist of glucose-6-phosphate dehydrogenase (G6PD)^44^, which catalyzes the first and committed step in the oxidative phase of the PPP^35^. AG1 treatment resulted in an increase in the proportion of TCF1 + cells with a corresponding increase in Eomes expression (Fig. 6A–6C) and reduced IFNγ production (Fig. 6B, 6D), consistent with PKM2^KO^ phenotypes and suggesting a skewing towards the TCF1^high^ progenitor population. We subsequently asked if PPP agonism induced this phenotype through a loss in glycolysis, which is known to result in inhibited effector differentiation^19^. To test this, we activated OT-I + Thy1.1 + T cells and subsequently co-cultured them with HKP1-ova-GFP cells until 6 days post-initial-stimulation. We then sorted the T cells from co-culture and treated them with either vehicle DMSO, AG1, or the hexokinase inhibitor 2-DG for 2 hours, then performed a glycolysis stress test. While acute treatment with 2-DG resulted in almost complete glycolytic shutdown, as expected, AG1 treatment had little effect on glycolysis, suggesting that PPP agonism may induce an altered differentiation state independent of glycolysis loss (Fig. 6E).

To evaluate the differential effects of these two different metabolic manipulations on CD8 + T cell differentiation, we bred a TCF1 eGFP reporter (Tcf7^GFP^, Reference^45^) mouse strain to the OT-I + strain, allowing us to track TCF1 expression in live cells. We activated the cells for 1 day, then initiated treatment with either DMSO, AG1, or 2-DG. We expanded the cells for 1 more day, then co-cultured them with HKP1-ova-GFP cells with continuing drug or vehicle treatment, passing cells every two days. At day 6 post-initial-stimulation, we sorted eGFP + and eGFP− cells from each condition and performed RNA sequencing. Both AG1 and 2-DG treatment resulted in increased proportions of eGFP + cells over the proportion emerging in the DMSO treated samples, indicating their abilities to impact differentiation (Fig. 6F–6G). 2-DG treatment resulted in a near complete loss of eGFP− cells, not permitting analysis of eGFP− cells under that treatment condition (Fig. 6F). Analyzing principal components and the top 1000 variable genes of eGFP + and eGFP− cells from the different treatments indicated large gene expression differences, both between samples based on TCF1 status and between eGFP + or eGFP− cells based on treatment, with 2-DG-eGFP + cells being the most transcriptionally distinct group (Fig. 6H–6I). Comparing eGFP + and eGFP− cells revealed expected gene expression differences, including increased Sell, Slamf6, Id3, Tcf7, and Nsg2 in eGFP + cells, and increased Gzma, Gzmb, Id2, Cd244a, and Havcr2 in eGFP− cells, validating the system and identifying key machinery conserved in TCF1 + cells across multiple metabolic modalities (Fig. 6J, **Extended Data Table 10**).

We subsequently asked how gene expression varied between eGFP + cells under different metabolic stimuli: baseline (via DMSO), PPP agonism (via AG1), or glucose blockade (via 2-DG). When compared to DMSO, both AG1 and 2-DG induced robust alterations to the transcriptional landscape, with 2-DG inducing more widespread differential gene expression (Fig. 6K, **Extended Data Table 11**). There was some overlap between treatments in genes over- or under-expressed compared with DMSO-treated eGFP + cells: 53.5% of up-regulated genes upon AG1 treatment were also up-regulated in 2-DG-treated cells, while 73.1% of down-regulated genes upon AG1 treatment were also down-regulated in 2-DG-treated cells (Fig. 6K). Given the larger disparity in gene expression between 2-DG treated samples and DMSO-eGFP + cells compared with the differential expression between AG1- and DMSO-treated eGFP + cells, this overlap in genes altered by treatment corresponded to only 9.1% of up-regulated genes and 25.5% of down-regulated genes in 2-DG treated samples (Fig. 6K). Further analyses comparing AG1-eGFP + and 2-DG-eGFP + samples supported a transcriptional landscape difference in TCF1 + cells generated by either PPP agonism or glucose blockade (Fig. 6L). Hallmark analysis showed an enrichment of proliferation (E2F targets, mitotic spindle) and inflammatory pathways (allograft rejection, complement, interferon gamma response) in AG1-eGFP + samples compared with enrichment of the unfolded protein response and hypoxia related pathways (mTORC1, hypoxia) in 2-DG-eGFP + samples (Fig. 6M). Taken together, these data indicate that PPP agonism and glucose blockade both induce TCF1 + populations, but with substantial differences in the transcriptional landscape.

Finally, we explored upstream regulators of gene expression, searching for conserved factors driving gene expression differences between PKM2^KO^ and PKM2^WT^ in our adoptive co-transfer experiment (**Extended Data Table 3**) and between AG1 and DMSO treatment in this *in vitro* co-culture experiment (**Extended Data Table 12**). Analysis showed a limited number of upstream regulators overlapping between these disparate experimental conditions, among them being Bach2 and Foxo1 (Fig. 6N, **Extended Data Table 13**). Both Bach2 and Foxo1 have been reported to be upstream inducers of TCF1 expression and regulators of memory formation and maintenance^46–50^, and had increased expression in both RNA sequencing datasets. We therefore examined their expression in our co-culture system by flow cytometry and found increased expression of both Foxo1 and Bach2 by both PKM2^KO^ and AG1 treatment at 4 days post-initial-stimulation compared to their respective controls, validating the sequencing results (Fig. 6O). Taken together, these data suggest shared expression of transcription factor machinery between PKM2^KO^ and PPP agonism previously reported to induce TCF1 expression.

### Pentose phosphate pathway agonism results in enhanced tumor control in combination with PD-1 checkpoint blockade.

**Figure 7:**
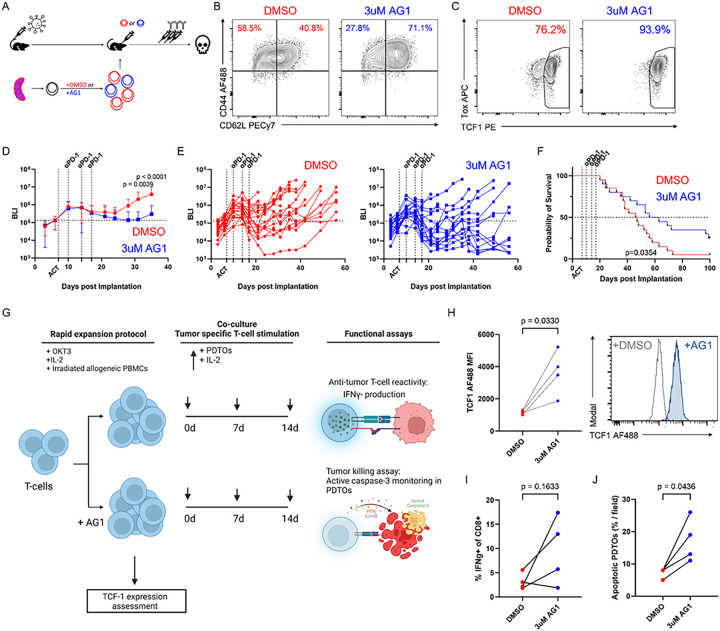
Pentose phosphate pathway agonism results in tumor control in murine and human model systems. **a-f**, OT-I+ Thy1.1+ CD8+ T cells were activated for 1 day, treated with either DMSO or 3 μM AG1 for 3 days, then adoptively transferred into lymphodepleted C57Bl/6 mice 7 days after orthotopic implantation of HKP1-ova-GFP tumors. 3 doses of anti-PD-1 were administered on days 10, 14, and 17 after tumor implantation. Phenotype of treated adoptively transferred T cells was evaluated, and tumor burden and overall survival monitored. **a,** Experimental schematic. **b-c,** Flow cytometric analyses of transferred T cells for CD44 and CD62L expression (b) and Tox and TCF1 expression (c). **d-e,** Bioluminescence imaging to measure average tumor burden (d) or tumor burden in individual mice (e) in mice which received DMSO-pretreated T cells (red) or AG1-pretreated T cells (blue). **f,** Overall mouse survival monitoring in mice which received DMSO-pretreated T cells (red) or AG1-pretreated T cells (blue). **g-j,** Patient derived tumor organoids (PDTOs) were generated from NSCLC samples from patients. Autologous T cells were expanded from matched peripheral blood mononuclear cells (PBMCs) by rapid expansion over two weeks with anti-human CD3 (OKT3), human IL-2, and irradiated allogenic PBMC feeder cells. During rapid expansion, T cells were treated with either DMSO control or 3μM glucose-6-phosphate dehydrogenase agonist AG1. After expansion, T cells were co-cultured with PDTOs and IL-2 to provide tumor-specific T cell stimulation in the presence. After two weeks, T cells were restimulated with fresh PDTOs, and T cell cytotoxic capacity tested by IFNγ production upon restimulation or tumor killing by PDTO cleaved caspase-3 activation. **g,** Experimental schematic. **h,** Quantification of TCF1 expression after rapid expansion as mean fluorescence intensity fold change over DMSO control, with representative histogram on the right. **i,** Quantification of IFNγ+ CD8+ T cells upon restimulation. **j,** Quantification of apoptotic PDTOs (CellTrace Far Red+ NucView488+ events) monitored over 12 hours. **Numbers:** (d-f) n=20 biological replicates per group, experiment repeated twice; (g-j) n=4 patients, 3 wells per patient. **Statistics:** (d) Two-way ANOVA, Šídák’s multiple comparisons with Grubbs’ outlier test with alpha = 0.0001; (f) Log-rank Mantel-Cox test; (h-j) paired t-test. **Abbreviations:** AF488, Alexa Fluor 488; PECy7, Phycoerythrin-Cyanine7; APC, Allophycocyanin; PE, Phycoerythrin.

As PPP agonism resulted in a TCF1^high^ population *in vitro*, similar to the effects of PKM2 knockout, we further tested if anti-tumor efficacy *in vivo* would be similarly enhanced in combination with anti-PD-1 therapy. Activated OT-I + T cells were pre-treated with either DMSO or AG1 for 3 days, adoptively transferred into cohorts of HKP1-ova-GFP mice, and subsequently administered 3 doses of anti-PD-1 antibody as described previously (Fig. 7A). Similar to results when co-cultured with tumor cells, pre-treatment with AG1 resulted in increased proportions of CD44 + CD62L + cells and TCF1 + cells prior to adoptive transfer (Fig. 7B-7C). Transfer of T cells pre-treated with AG1 resulted in better tumor control (Fig. 7D-7E) and overall survival (Fig. 7F) in combination with anti-PD-1 than T cells pre-treated with DMSO. Taken together, these results demonstrate that PKM2 loss results in amplification of PPP activity, and that PPP agonism phenocopies the results of PKM2 loss both *in vitro* and *in vivo*, with enhanced generation of TCF1^high^ progenitor cells responsive to PD-1 blockade resulting in better anti-tumor immunity.

### Pentose phosphate pathway agonism promotes anti-tumor immunity in ex vivo immunocompetent human patient-derived tumor organoids.

Our studies in mouse T cells showed a potent ability of PPP agonism to induce a TCF1 + progenitor state and combine with checkpoint blockade to yield a significant tumor control and survival benefit. We therefore tested the effects of AG1 in a human immunocompetent patient-derived tumor organoid system^51,52^ (Fig. 7G). Following standard methodologies^51,52^, we generated patient-derived tumor organoids (PDTOs) from NSCLC patient specimens. We rapidly expanded^53^ T cells from autologous peripheral blood mononuclear cells (PBMCs) donated by the patients for two weeks, either in the presence or absence of AG1 (Fig. 7G), then assessed TCF1 expression. Similar to our findings in mice, AG1 treatment significantly increased TCF1 expression in samples from 4 out of 4 patients (Fig. 7H). Expanded T cells were co-cultured with autologous PDTOs for two weeks, then restimulated for analysis for tumor reactivity by IFNγ production and tumor killing by cleaved caspase-3 production in tumor organoids. While IFNγ production was variable, AG1-treated T cells produced higher levels of IFNγ than their DMSO-treated counterparts in samples from 3 out of 4 patients (Fig. 7I). Importantly, AG1-treated T cells consistently induced significantly higher proportions of apoptotic organoids than their DMSO-treated counterparts, with a consistent phenotype between all 4 patient samples (Fig. 7J). Taken together, these data indicate that agonism of the pentose phosphate pathway may augment the anti-tumor capabilities of T cells in humans.

## Discussion

Our global gene expression profiling of treatment-naïve and PD-1 blockade-treated HKP1 tumors identified metabolic regulatory pathways in intratumoral CD8 + T cells, consistent with the critical roles of metabolism in regulating T cell fate and function^13–16^. Of the metabolic alterations identified, glycolysis was enriched in T cells of progressing tumors. Glycolytic function is critical for optimal effector activity of T cells^13,14,54–58^. Our shRNA screen targeting a majority of differentially expressed glycolytic enzymes in intratumoral T cells displayed a spectrum of phenotypes, including loss of effector cytokine production or proliferative status, consistent with recent reports^17,19,54–62^, whereas knockdown of other glycolytic genes resulted in upregulation of checkpoint proteins, implicating a T cell dysfunction phenotype. Intriguingly, the screen showed that loss of metabolic regulator PKM2 induced a progenitor-exhaustion like state, associated with increased expression of the transcription factor TCF1. This altered differentiation of activated T cells with PKM2 loss generated a central memory-like phenotype *in vivo*, which combined with anti-PD-1 to yield significant tumor control and improved overall survival. Further mechanistic explorations detailed a decreased glycolytic metabolism and an increased utilization of the pentose phosphate pathway (PPP). Subsequent exploration of the ramifications of increased PPP activity revealed its capacity to induce a TCF1^high^ transcriptional state independent of and distinct from glycolytic blockade, and therapeutic utility controlling tumors both in an *in vivo* mouse lung cancer model in combination with anti-PD-1, and in an *ex vivo* immunocompetent human patient-derived tumor organoid system. These data describe a new metabolic reprogramming that contributes to a progenitor-like T cell state amenable to therapeutic checkpoint blockade.

PKM2 is expressed in various cell types, and both normal and disease states. Several reports have investigated PKM2 in CD4 + T cells in different non-tumor inflammatory contexts, indicating a role for PKM2 in glycolysis and a resultant activation phenotype^31–34,63^. One study reported metabolic data demonstrating a dampening of glycolysis and pentose phosphate pathway activity upon PKM2 loss^63^. Other studies have focused on PKM2’s transcriptional regulation during Th differentiation^31,34^, and effects of pharmacological inhibition or activation on Th polarization^32–34^. Our results in CD8 + T cells critically diverge from these findings. First, in contrast to effects of its loss in CD4 + T cells, we observe enhanced PPP activity upon PKM2 knockout in CD8 + T cells, suggesting a different metabolic compensation. Second, PKM2 plays an important role in the nucleus of CD4 + T cells upon their activation and during differentiation; in contrast, we observe little to no accumulation in the nucleus, suggesting most if not all PKM2 is cytoplasmic. Furthermore, knockout of PKM2 in naïve CD8 + T cells results in upregulation of PKM1 upon activation and normal differentiation to effector CD8 + T cells, suggesting that metabolic pyruvate kinase activity is sufficient for this process (data not shown). While we hesitate to completely exclude the other regulatory effects of PKM2, its subcellular localization, the relative abundance of cytoplasmic and nuclear PKM2, and the ability of PKM1 to substitute and allow normal effector differentiation suggest a markedly different functionality for PKM2 in CD8 + T cells compared with that delineated in CD4 + T cells.

Our steady-state metabolomics and 1,2 ^13^C glucose carbon tracing data demonstrate that PKM2 loss in CD8 + T cells resulted in accumulation of PPP metabolites. The presence of increased amounts of alternatively labelled isotopes of metabolites in PKM2^KO^ T cells indicated more cycles of PPP activity^36,37^ compared with that observed in PKM2^WT^ cells. Small molecule agonism of glucose 6-phosphate dehydrogenase, the first enzyme in the oxidative PPP, to induce the altered metabolic state observed upon PKM2 loss resulted in a similar altered differentiation towards a TCF1 + progenitor-like state. These data suggest that PKM2 loss may lead to this TCF1 + progenitor-like phenotype by amplifying PPP activity. A reasonable experiment to test this hypothesis would be to abrogate PPP activity and examine the effects of PKM2 manipulation on differentiation. However, this experiment is complicated by the reliance of CD8 + T cell differentiation on the PPP, which has been previously reported^41,43^. Based on those data, we tested the effects of inhibition of the PPP using small molecule inhibitors targeting different enzymes in the pathway: G6PDi-1, inhibiting glucose 6-phosphate dehydrogenase; 6-aminonicotinamide (6-AN), inhibiting 6-phosphogluconate dehydrogenase; and oxythiamine, inhibiting transketolase. We evaluated a variety of doses and treatment windows for these inhibitors, and consistently found with both short and long-term treatments that both G6PDi-1 and 6-AN, which impact the oxidative PPP, resulted in significantly reduced effector T cell populations in both PKM2^WT^ and PKM2^KO^ T cells, while blocking the non-oxidative phase of the PPP with oxythiamine had a negligible effect (**Extended Data Fig. 10**). Furthermore, long-term blockade of the oxidative PPP resulted in precipitous cell loss, suggesting a failure to either differentiate or thrive. In our hands, treatment of equivalent initial numbers of cells resulted in a cell yield in the G6PDi-1 group of only 20.7% of the DMSO-treated cell number at 1 day after treatment initiation. This loss continued, with G6PDi-1-treated cell numbers falling to 7.9% and 2.9% of timepoint-matched DMSO-treated control cell numbers after 3 days and 5 days respectively of treatment. These data are consistent with the previous studies^41,43^ showing negative effects of PPP inhibition on CD8 + T cell differentiation, and along with our findings using the PPP agonist AG1 and the hexokinase inhibitor 2-DG, suggest that balanced glycolysis and pentose phosphate pathway activity is necessary for robust effector differentiation.

Loss of PKM2 in CD8 + T cells resulted in a TCF1 + progenitor-like state. Similar phenotypes have also been observed following inhibition of other glycolytic enzymes including hexokinase (HK)^19^ and lactate dehydrogenase (LDH)^17^. However, this progenitor- or memory-like state induced by disruption of PKM2, HK, or LDH activity is mediated by disparate molecular mechanisms: PKM2 loss results in enhanced PPP activity, while pharmacological HK inhibition results in amplification of fatty acid oxidation (FAO)^19^ and transient LDH inhibition results in increased tricarboxylic acid cycle (TCA) activity^17^. This differential metabolic rerouting suggests multiple redundant mechanisms converge on a TCF1 + differentiation state similar in some crucial respects, including TCF1 and cytokine expression patterns and cell proliferation, and display overlap with progenitor-exhausted populations described in the literature^5–8,64−66^. However, the utilization of divergent metabolic pathways in these cells suggested there may be corresponding transcriptional heterogeneity. Our sequencing experiments using a TCF1 reporter to sort viable TCF1 + and TCF1^−^ cells differentiated under PPP agonism via AG1 or glucose blockade via 2-DG demonstrated significant differences in the transcriptional landscape of TCF1 + cells. AG1-treated cells were more proliferative, more transcriptionally active, and with more inflammatory signatures, while 2-DG-treated cells had significantly higher unfolded protein response and hypoxia signatures. These differences require further exploration, but may in turn be leveraged therapeutically, allowing selection of different metabolic modalities and cognate transcriptional profiles for use in different tissue, disease, or disease-stage contexts.

Our data demonstrate that PKM2 loss resulted in accumulation of glycolytic intermediates and increased PPP activity. Agonism of G6PD activity phenocopied PKM2 loss. This suggested a conserved mechanism could be used in both contexts to result in generation of the TCF1 + progenitor populations. Upstream regulator analysis comparing RNA sequencing data from PKM2^KO^ and PKM2^WT^ T cells isolated back from mouse tumors after adoptive co-transfers and from AG1 and DMSO treated T cells isolated after *in vitro* co-culture with tumor cells demonstrated conserved Foxo1 and Bach2 signatures. Importantly, expression of both Foxo1 and Bach2 was increased by PKM2 loss or AG1 treatment at 4 days post-initial-stimulation, suggesting that these factors may play a mechanistic role downstream of PPP activity inducing TCF1. Interestingly, while a Bach2 upstream regulator signature was observed in TCF1 + cells induced by 2-DG treatment compared with TCF1 + cells in the DMSO control, a Foxo1 signature was not observed (**Extended Data Table 13**), suggesting differential transcription factor usage for generation and maintenance of this distinct TCF1 + state. Foxo1 and Bach2 are well-known regulators of stem and memory characteristics and regulate TCF1 expression^46–50^. Ongoing experiments are exploring how these two proteins and other core memory/progenitor-like machinery are directly regulated by PPP activity. One intriguing possibility is through epigenetic control. NADPH, an important bioactive reducing agent produced in the oxidative PPP^35^, has been recently shown to bind to HDAC3, resulting in an inability to interact with its co-activators NCOR1 and NCOR2^67^. These data suggest that PPP activity may impact epigenetic control of T cell differentiation. Indeed, recent work has shown that HDAC3 controls CD8 + T cell cytotoxicity programs^68^, while other studies have shown important roles for EZH2^69^ and SUV39H1^70^ in controlling T cell differentiation and effector function. Future experiments will explore the ramifications of amplified PPP activity on transcriptional and epigenetic control of T cell fate.

Finally, our work raises interesting possibilities for the treatment of human disease. PKM2 displayed similar expression patterns, and its knockout had similar phenotypic results in tumor-specific T cells in both a non-small cell lung cancer model and a melanoma model, suggesting a conserved mechanistic response regardless of tumor type, although with potential differences in kinetics and magnitude. Using both flow cytometry and RNA sequencing, we observed that anti-PD-1 treatment resulted in induction of similar phenotypes in PKM2^WT^ and PKM2^KO^ T cells, including greater proliferation and a transition to a more effector-like phenotype, suggesting that the machinery for response to the therapy was largely intact. Loss of PKM2 did however result in a sustained higher proportion of a memory-like cellular compartment, with a limited terminal effector response, which has been shown to be important for superior long-term protection^71^. Treatment with the G6PD agonist AG1 demonstrated that PPP amplification resulted in a similar phenotypic skewing to a TCF1 + progenitor-like state in co-culture with tumor cells, and *in vitro* pre-treatment of tumor-specific T cells with AG1 resulted in enhanced tumor control upon adoptive transfer in combination with PD-1 blockade. Using a human immunocompetent patient-derived tumor organoid (PDTO) platform, we demonstrated that treatment with AG1 during T cell rapid expansion from autologous PBMCs resulted in elevated TCF1 expression, mimicking our mouse data. After culture of these expanded T cells with autologous PDTOs to provide tumor-specific T cell stimulation, assays to assess T cell reactivity and tumor killing potency displayed elevated T cell effector function in AG1-treated cells. Together, these suggest that PPP amplification may be a useful therapeutic modality in adoptive cell transfer immunotherapy approaches, and may synergize with PD-1 blockade to provide durable clinical benefits.

## Methods

### Animal work.

All animal work was performed in accordance with an animal protocol approved by the institutional Animal Care and Use Committee at WCMC (Protocol number 0806–762A). Female C57BL/6J (catalogue number 000664) mice were purchased from The Jackson Laboratory (Bar Harbor, Maine). OT-I mice^25^ on C57BL/6J (C57BL/6-Tg(TcraTcrb)1100Mjb/J, catalogue number 003831) and Thy1.1^+/+^ mice on C57BL/6J (B6.PL-Thy1^a^/CyJ, catalogue number 000406) were purchased from The Jackson Laboratory and bred in-house to produce OT-I^+^/Thy1.1^+/+^ and OT-I^+^/Thy1.1^+/−^ mice. Tcf7^GFP^ mice^45^ on C57BL/6J (B6(Cg)-*Tcf7*^tm1Hhx^/J, catalogue number 030909) were purchased from The Jackson Laboratory and bred in-house to OT-I^+^/Thy1.1^+/+^ and OT-I^+^/Thy1.1^+/−^ to produce Tcf7GFP^+^/OT-I^+^/Thy1.1^+/+^, Tcf7GFP^+^/OT-I^+^/Thy1.1^+/−^, and Tcf7GFP^+^OT-I^+^/Thy1.1^−/−^ mice.

### Patient sample collection and use.

All patient samples were obtained following informed consent from the Cardiothoracic Surgery Department, Weill Cornell Medical College (WCMC, New York, NY). Specimens were collected after obtaining written informed consent prior to undergoing any study-specific procedures in accordance with the Declaration of Helsinki. Patient identity for pathological specimens remained anonymous in the context of this study. Patient sample collection was approved by the Institutional Review Board of Weill Cornell Medical College; Thoracic Surgery Biobank Protocol Number 1008011221.

### Culturing of cell lines and primary T cells.

mCherry-Luciferase expressing HKP1 lung cancer cells^20^ and B16F10 cells purchased from ATCC (catalogue number CRL-6475) were retrovirally transduced with pMFG-Ova-N4-EGFP (a generous gift from Dr. Andrea Schietinger and Dr. Mary Philip) for expression of the Ova_257 – 264_ immunogenic peptide SIINFEKL along with Enhanced Green Fluorescent Protein to generate the HKP1-ova-GFP and B16F10-ova-GFP cells utilized in this study. Successfully transduced cells were selected by fluorescence-activated cell sorting (FACS) and validated by overnight stimulation with 20ng/mL IFNγ and subsequent flow cytometric analysis for EGFP expression and presentation of SIINFEKL on MHC class I. HKP1 and HKP1-ova-GFP cell lines were maintained in DMEM (Corning) supplemented with 10% Fetal bovine serum (FBS, Atlanta Biologicals), 100 U/mL penicillin with 100μg/mL streptomycin (Corning), and 2mM L-glutamine (Corning). B16F10, B16F10-ova-GFP, and HEK293T cell lines were maintained in DMEM supplemented with 10% FBS and 100 U/mL penicillin with 100μg/mL streptomycin. Platinum-E Retroviral Packaging Cells (Plat-E) were a generous gift from Dr. Morgan Huse, and were cultured in DMEM supplemented with 10% FBS (Atlanta Biologicals), 100 U/mL penicillin with 100μg/mL streptomycin (Corning), 2mM GlutaMAX (Gibco), 10mM HEPES Buffer (Corning), and 50μM β-mercaptoethanol (Sigma). Primary mouse T cells were cultured in Advanced RPMI (RPMI-1640 with non-essential amino acids and 110mg/L sodium pyruvate; Gibco) supplemented with 10% FBS (Atlanta Biologicals), 100 U/mL penicillin with 100μg/mL streptomycin (Corning), 2mM GlutaMAX (Gibco), 10mM HEPES (Corning), and 50μM β-mercaptoethanol (Sigma), designated Complete T Cell Media. For glucose isotope labelling experiments, T cells were sorted from co-culture into RPMI 1640 Medium with L-Glutamine and without Glucose (Gibco) supplemented with 10% heat-inactivated dialyzed (12–14kD) FBS (Atlanta Biologicals), insulin-transferrin-selenium-ethanolamine (ITS-X, Gibco), 1mM sodium pyruvate (Gibco), 100 U/mL penicillin with 100μg/mL streptomycin (Corning), 50μM β-mercaptoethanol (Sigma), and 50U/mL IL-2 (Tracer Sort Media). T cells were subsequently labelled by culturing in the Tracer Sort Media supplemented with 2000mg/L 1,2–13C2 Glucose (Cambridge Isotope Laboratories), designated Labelling Media. All cells were cultured at 37°C, 5% CO_2_ unless otherwise specified.

### Orthotopic HKP1 and HKP1-ova-GFP models.

150,000 HKP1 cells or 250,000 HKP1-ova-GFP cells suspended in sterile PBS were administered via the tail vein into syngeneic female 8-week-old C57BL/6J mice. Tumor growth in vivo was evaluated twice weekly via bioluminescence imaging (BLI) using a Xenogen IVIS system. To divide mice into cohorts for different treatments, BLI data were collected 1 day prior to treatment and mice were grouped such that similar mean tumor burdens were present in each treatment cohort. For treatment with anti-PD-1 or IgG2a, mice were injected intraperitoneally with 250μg of anti-PD-1 or IgG2a control on days 10, 13, and 17 post-tumor implantation. For all survival studies, mouse tumor burden was evaluated bi-weekly, and mice were euthanized when tumor burden reached humane endpoints.

### Orthotopic B16F10-ova-GFP model.

Prior to the administration of cells, mice were anesthetized with isoflurane and fur removed on the dorsal right flank. Depilatory cream for fur removal was left on the flank for 30–60 seconds and promptly wiped off using warm water and gauze. 500,000 B16F10-ova-GFP cells suspended in 100 μL sterile PBS were administered via subcutaneous injection into syngeneic female 8-week-old C57BL/6J mice. Tumor growth in vivo was evaluated twice weekly via caliper measurements (Fisherbrand Traceable Digital Caliper) and tumor volumes was calculated with the formula *V* = *L(W^2^)/2*, where L and W are tumor length and width (mm), respectively.

### Retrovirus production.

Retroviruses were packaged by calcium-phosphate transfection of packaging cells similar to previously published methodologies^72^. For retrovirus production with the pMFG-Ova-N4-EGFP construct, HEK293T cells were plated, treated with chloroquine, then transfected with pMFG-Ova-N4-EGFP and the packaging plasmid Phoenix. Cells were incubated for 10–14 hours at 37°C, 5% CO_2_, and then media exchanged for fresh antibiotic-free media with sodium butyrate and cells moved to a tissue culture incubator at 32°C, 5% CO_2_. Virus was collected at 48 hours and 72 hours post-transfection.

For retrovirus production with shRNA plasmids, Plat-E cells were plated, treated with chloroquine, then transfected with shRNA plasmid DNA, packaging plasmid pCL-Eco (Addgene), and siRNA targeting DGCR-8/Pasha (IDT).

Cells were incubated for 10–14 hours at 37°C, 5% CO_2_, and then media exchanged for fresh antibiotic-free media with sodium butyrate and cells moved to a tissue culture incubator at 32°C, 5% CO_2_. Virus was collected at 48 hours and 72 hours post-transfection.

### T cell isolation and stimulation.

One day prior to T cell isolation, plates were coated sequentially with biotinylated poly-L-lysine (Sigma, Thermo Scientific), Streptavidin-Plus (Prozyme), and finally with biotin anti-CD3ε and biotin anti-CD28. Coated plates were wrapped in parafilm to prevent evaporation and incubated overnight at 4°C.

On the day of T cell isolation, mice of specified genotypes were euthanized, and spleens dissected and ground through a 40μm filter (Cell Strainer, 40 micron, Nylon; Falcon) into Complete T Cell Media. Resultant single-cell suspensions were pelleted, red blood cells lysed with ACK Lysing Buffer (Quality Biological), and pellets washed and resuspended in FACS Buffer (PBS supplemented with 0.5% BSA and 2mM EDTA). Untouched CD8^+^ T cells were separated using the Militenyi Magnetic Activated Cell Sorting (MACS) system, and efficiency and purity of isolation confirmed by flow cytometry. Untouched CD8^+^ T cells were resuspended in Complete T Cell Media supplemented with 50U/mL IL-2 and plated on anti-CD3ε and anti-CD28 coated plates at approximately 0.75×10^6^ cells/mL.

### T cell shRNA retroviral transduction and sorting.

Antigen-specific splenic CD8^+^ T cells were isolated and stimulated with plate-bound anti-CD3ε and anti-CD28 (2μg/mL each) as described above. 18 hours after plating, media was replaced with retrovirus-containing media supplemented with 4μg/mL polybrene (hexadimethrine bromide, Sigma-Aldrich) and 50U/mL IL-2. T cells were spin-transduced at 1000 × g for 90 minutes at 30°C, and subsequently moved to a 32°C incubator for 4 hours. Retrovirus-containing media was replaced with fresh Complete T Cell Media supplemented with 50U/mL IL-2. Cells were returned to the 32°C incubator for another 7.5 hours, then transferred to a 37°C incubator. T cells were sorted approximately 48–50 hours after initial plating on a Becton-Dickinson FACS Aria II sorter for successfully transfected viable T cells (DAPI^−^ ZsGreen^+^).

### T cell CRISPR/Cas9 ribonucleoprotein (RNP) electroporation and sorting.

Antigen-specific splenic CD8^+^ T cells were isolated and stimulated as above. 24 hours after plating, T cells were transfected with CRISPR/Cas9 ribonucleoprotein via electroporation utilizing the Neon Transfection System (Thermo Fisher Scientific) and ATTO-conjugated tracrRNA, crRNAs, and Cas9 protein purchased from IDT similar to previously published methodologies^73^. RNPs were formed by mixing Cas9 and duplexed ATTO-tracrRNA:crRNA at a 1:1.2 molar ratio (1:1 volume ratio), and incubated at room temperature for 20 minutes. Activated viable T cells were counted and resuspended in Neon Buffer T, then mixed with RNP and electroporation enhancer. The mixture was pipetted using the Neon pipette and Neon tips and the pipette plugged into position in the Neon transfection device. Electroporation was performed at 3 pulses, 10ms pulse width, 1600V. T cells were subsequently rested in Complete T Cell Media with 50U/mL IL-2 for at least 2 hours, then successfully transfected viable T cells (DAPI^−^ ATTO550^+^) were sorted on a Becton-Dickinson FACS Aria II, and subsequently expanded on anti-CD3ε and anti-CD28 (2μg/mL each) coated plates in Complete T Cell Media with 50U/mL IL-2 for 24 hours. Knockout efficiency was validated 72 hours later by flow cytometry.

### T cell/tumor cell co-culture and staining.

For baseline co-culture dynamics and phenotypes, antigen-specific CD8^+^ T cells were activated either via antigen presentation from splenic antigen-presenting cells (APCs) or via stimulation with plate-bound anti-CD3ε and anti-CD28 as above. Both methodologies yielded similar results. For antigen presentation from APCs, red blood cell lysed single cell suspensions were made from spleens from donor mice, cultured with 1μg/mL Ova_257 – 264_ peptide (InvivoGen), and plated at 2mL per well in 12 well tissue culture plates. Two days later, activated antigen-specific CD8^+^ T cells were isolated from the culture using the Militenyi MACS system for untouched CD8^+^ T cell isolation. The alternative activation method utilizing plate-bound anti-CD3ε and anti-CD28 was performed as described above: untouched CD8^+^ T cell isolation via Militenyi MACS sorting, and culturing on plate-bound anti-CD3ε and anti-CD28 at 2μg/mL each in Complete T Cell Media with 50U/mL IL-2. These cells were also cultured for two days. In both experiments, T cells were subsequently either co-cultured with tumor cells or passed to plates coated with anti-CD3ε and anti-CD28 at 2μg/mL each. For co-cultures, T cells were mixed with antigen-expressing tumor cells (HKP1-ova-GFP) at a 5:1 effector:target (E:T, T cell:tumor cell) ratio and plated in Complete T Cell Media with 50U/mL IL-2, a modification of previously published methodologies^74^. 5×10^5^ T cells and 1×10^5^ tumor cells were plated per well in a 12 well plate for flow cytometry staining experiments, 1×10^6^ T cells and 2×10^5^ tumor cells for Seahorse and steady-state metabolomic experiments, and 4×10^5^ T cells and 8×10^4^ tumor cells per well for isotope tracing experiments. T cells were passed every other day, and either plated on fresh anti-CD3ε and anti-CD28 coated plates or mixed 5:1 E:T in Complete T Cell Media with 50U/mL IL-2. Cells were harvested twice weekly for analysis.

In experiments with genetic manipulations, T cells were modified as described above, and co-cultures or sequential plate-bound stimulations initiated 48 hours after initial T cell activation. In experiments with treatment with the G6PD agonist AG1, T cells were isolated and activated on anti-CD3ε and anti-CD28 coated plates (2μg/mL each) as described above, cultured in Complete T Cell Media with 50U/mL IL-2 for 24 hours, then treated with either 3μM AG1 or an equivalent volume of DMSO and cultured for an additional 24 hours. Co-cultures with tumor cells were then initiated as above and treatment continued with 3μM AG1 or an equivalent volume of DMSO in Complete T Cell Media with 50U/mL IL-2 for the duration of the experiment.

In experiments with the TCF1 eGFP reporter (Tcf7^GFP^) mice, T cells were isolated from Tcf7^GFP+^ OT-I^+^ mice and activated on anti-CD3ε and anti-CD28 coated plates (2μg/mL each) as described above, cultured in Complete T Cell Media with 50U/mL IL-2 for 24 hours, then treated with 3μM AG1, 2mM 2-deoxyglucose (2-DG), or an equivalent volume of DMSO and cultured for an additional 24 hours. Co-cultures with tumor cells were then initiated as above and treatment continued with 3μM AG1, 2mM 2-DG, or an equivalent volume of DMSO in Complete T Cell Media with 50U/mL IL-2 for the duration of the experiment, being passed every two days and replenished with drug or DMSO as before.

In experiments with treatment with the Pentose Phosphate Pathway inhibitors G6PDi-1, 6-aminonicotinamide, and oxythiamine, T cells were isolated and activated on anti-CD3ε and anti-CD28 coated plates (2μg/mL each) as described above, cultured in Complete T Cell Media with 50U/mL IL-2 for 2 days, then co-cultured with tumor cells as above for 2 days, then co-cultures passed and drugs or an equivalent volume of DMSO added at indicated doses in Complete T Cell Media with 50U/mL IL-2 for the last 2 days.

For experiments requiring purified T cell populations after long-term culture, cells were harvested by pipetting suspensions from the plates, filtering through a 70μm filter (Cell Strainer, 70 micron, Nylon; Falcon), washing with PBS, then staining for CD8β and Thy1.1. Samples were dyed with DAPI at 0.2μg/mL, and sorted on a Becton-Dickinson FACS Aria II sorter for DAPI^−^ CD8β^+^ Thy1.1^+^ cells. For experiments with the TCF1 eGFP reporter (Tcf7^GFP^), cells were harvested, filtered, and washed, and stained for CD8β and Thy1.2. Samples were dyed with DAPI at 0.2μg/mL, and sorted on a Becton-Dickinson FACS Aria II sorter for DAPI^−^ CD8β ^+^ Thy1.2^+^ eGFP + or DAPI^−^ CD8β ^+^ Thy1.2^+^ eGFP− cells.

### T cell subcellular fractionation and Western blotting.

To examine subcellular localization of PKM isoforms, T cells were harvested, activated, genetically modified, and co-cultured with tumor cells as described above. At day 4 post-initial-stimulation, CD8 + Thy1.1 + T cells were isolated from co-culture using the EasySep Mouse CD90.1 Positive Selection Kit from STEMCELL Technologies according to the manufacturer’s instructions. Briefly, co-cultures were aspirated, filtered through 40 micron filters, resuspended at 1×10^8^ cells/mL, transferred into 5 mL polystyrene round-bottom tubes, and then selection cocktail was added to sample at 50μL/mL sample, and mixed and incubated for 3 minutes at room temperature. RapidSpheres were then added to the sample at 40μL/mL sample, and mixed and incubated for 3 minutes at room temperature. Samples were topped up to 2.5mL in FACS Buffer, gently pipetted, and placed on an EasyEights separation magnet for 10 minutes at room temperature. Supernatant was subsequently pipetted off, and the washing with FACS Buffer and incubation on the magnet performed twice more for a total of 3 × 10 minute separations. Cells were then aliquoted and protein subsequently extracted.

Cytoplasmic, nuclear, and whole cell extracts of the isolated T cells were collected using the PARIS (Protein and RNA Isolation System) Kit from Thermo Fisher according to the manufacturer’s protocol. Briefly, cells were isolated from co-culture, pelleted, washed once with PBS, and re-pelleted. Aliquots for whole cell extracts were resuspended in Cell Disruption Buffer supplemented with protease (cOmplete protease inhibitor cocktail tablets, Roche) and phosphatase (PhosSTOP phosphatase inhibitor cocktail tablets, Roche) inhibitors, vortexed for 1 minute, and incubated on ice for 10 minutes before being centrifuged at max speed for 2 minutes at 4°C, and supernatant aspirated and stored at −80°C until use. Cell aliquots for cytoplasmic and nuclear extract generation were resuspended by gently pipetting 5 times in Cell Fractionation Buffer supplemented with protease and phosphatase inhibitors, and incubated on ice for 10 minutes. Cells were then centrifuged at 500×g for 5 minutes at 4°C, and the supernatant (the cytoplasmic extract) was aspirated and stored at −80°C until use. The remaining pellet was washed again with the Cell Fractionation Buffer supplemented with protease and phosphatase inhibitors, centrifuged at 500×g for 2 minutes at 4°C, supernatant discarded, and pellet resuspended in Cell Disruption Buffer supplemented with protease and phosphatase inhibitors. The resuspended pellet was vortexed for 1 minute and pipetted for 2 minutes, then incubated on ice for 10 minutes before being centrifuged at max speed for 2 minutes at 4°C, and supernatant (the nuclear extract) aspirated and stored at −80°C until use.

Western blotting was performed using standard methodologies. Briefly, protein content from extracts was determined by a Pierce BCA Protein Assay (Thermo Scientific), and equivalent amounts of protein were mixed with loading dye (NuPAGE LDS Sample Buffer), boiled at 95°C for 5 minutes, then loaded on 4–15% precast gels (Mini-PROTEAN TGX Precast Protein Gels, Bio-Rad), stacked at 80V for 20–30 minutes, then run for 120V for another 20–30 minutes. Protein was transferred wet onto PVDF membranes at 250mA for 1.5 hours, then membranes were washed briefly in TBS-T, blocked with 5% milk in TBS-T at room temperature for 1 hour under gentle agitation, briefly washed in 5% BSA in TBS-T, then incubated with primary antibodies at indicated concentrations in indicated diluents overnight at 4°C under gentle agitation. After at least 16 hours, membranes were washed 3 times for 5 minutes with TBS-T under gentle agitation, then incubated with secondary antibody diluted in 5% milk in TBS-T at room temperature for 1 hour under gentle agitation. Membranes were then washed 3 times for 5 minutes with TBS-T at slightly faster agitation, then membranes were developed using Amersham ECL Prime Western Blotting Detection Reagent (GE Healthcare) and imaged using a Bio-Rad ChemiDoc XRS + with Image Lab Software. Where necessary, blots were then washed once for 5 minutes with TBS-T under gentle agitation, stripped for 1 hour at room temperature under gentle agitation using Restore Western Blot Stripping Buffer (Thermo Scientific), then blocked, incubated with primary antibody, washed, incubated with secondary antibody, washed, and developed and imaged as before.

### Flow cytometry staining.

For surface stains, samples were incubated with a fixable live/dead viability dye (Zombie Aqua, Biolegend), stained with primary antibodies in the presence of Fc blockers, washed with FACS Buffer, fixed with 1% formaldehyde, washed with FACS Buffer, and resuspended in FACS Buffer.

For intracellular transcription factor stains, samples were incubated with a fixable live/dead viability dye, stained with primary antibodies in the presence of Fc blockers, washed with FACS Buffer, fixed with Fixation/Permeabilization Buffer (eBioscience), washed with Permeabilization Buffer (eBioscience), stained with primary antibodies for intracellular markers in Permeabilization Buffer, washed with Permeabilization Buffer, and resuspended in FACS Buffer.

For intracellular stains for cytokines and effector proteins, samples were Golgi blocked via treatment with Brefeldin A (Biolegend) and Monensin (Biolegend) for 5 hours in Complete T Cell Media at 37°C in a humidified incubator. Samples were subsequently stained using the same methods as for transcription factors.

For intracellular stains requiring methanol permeabilization, samples were incubated with a fixable live/dead viability dye, washed with PBS, fixed with 4% formaldehyde, washed with PBS, permeabilized with 90% methanol, washed with PBS, stained with primary antibodies in the presence of Fc blockers, washed with FACS Buffer, and resuspended in FACS Buffer.

After staining, all samples were covered in aluminum foil and stored at 4°C until analysis (less than 24 hours later). Data were acquired on a Becton-Dickinson LSRFortessa, a Becton-Dickinson LSR II, or a Becton-Dickinson Symphony A5 and analyzed with FlowJo 10 (FlowJo, LLC).

### Seahorse extracellular flux analyses.

Glycolytic stress tests were performed on sorted T cells from co-cultures using the Agilent Seahorse XF Glycolysis Stress Test Kit (Agilent, 103020–100). Briefly, antigen-specific T cells were isolated, activated, genetically modified by CRISPR/Cas9, and co-cultured, then resuspended, stained for DAPI, CD8β, and Thy1.1, and viable cells sorted into Complete T cell Media as described above at 6 days post-initial-stimulation. T cells were washed with pre-warmed assay media (Seahorse XF DMEM Medium, pH 7.4, 2mM glutamine), and resuspended at 1.25–1.5×10^5^ cells per 50μL assay media. Resuspended T cells were plated on Seahorse XF96 Cell Culture Microplates coated with 22.4 μg/mL Cell-Tak (Corning) by centrifugation with no brake, put into a 37°C non-CO_2_ incubator for 30 minutes, supplemented with an additional 130μL of assay media, and returned to the incubator. Sensor cartridges in utility plates were pre-hydrated with Seahorse XF Calibrant in a 37°C non-CO_2_ incubator, and appropriate ports loaded: Port A-Glucose (10X port concentration: 100mM), Port B-Oligomycin (10X port concentration: 10μM, Port C-2-DG (10X port concentration: 500mM). Assays were initiated in the XF software, sensor cartridges loaded and calibrated, then cell culture microplates loaded and assays run on an XFe96 Extracellular Flux Analyzer (Agilent). DNA content was measured for Seahorse data normalization post-run by Hoechst 33342 staining and fluorescence read at 361/486nm. A standard curve using reference DNA stained simultaneously allowed for calculation of DNA content in ng and was used to normalize Seahorse data.

For experiments testing the acute effects of drugs on glycolysis, antigen-specific T cells were isolated, activated, and co-cultured with HKP1-ova-GFP as described previously, then resuspended, stained for DAPI, CD8β, and Thy1.1, and viable cells sorted into Complete T cell Media as described above at 6 days post-initial-stimulation. Sorted cells were then washed with pre-warmed assay media (Seahorse XF DMEM Medium, pH 7.4, 2mM glutamine), and resuspended at 1.5×10^5^ cells per 50μL assay media supplemented with indicated doses of drugs. Resuspended T cells were plated on Seahorse XF96 Cell Culture Microplates coated with 22.4 μg/mL Cell-Tak (Corning) by centrifugation with no brake, put into a 37°C non-CO_2_ incubator for 30 minutes, supplemented with an additional 130μL of assay media supplemented with drugs, and returned to the incubator for an additional 1.5 hours. Glycolytic stress tests were subsequently performed as described above.

### T cell adoptive transfer.

For adoptive transfer of naïve antigen-specific CD8^+^ T cells, untouched splenic CD8^+^ T cells from donor mice were isolated as above using the Militenyi MACS system. Cells were then washed twice with PBS and resuspended at 1×10^6^ cells/mL in sterile PBS. Syngeneic female 8-week-old C57BL/6J recipient mice were anesthetized with isoflurane, and 5×10^5^ cells in 50μL PBS were transferred retro-orbitally.

For adoptive transfer of *in vitro* activated or genetically modified or drug treated antigen-specific CD8^+^ T cells, syngeneic female 8-week-old C57BL/6J received tumor implantation as described above 7 days prior to adoptive transfer. Tumor-bearing mice received 5 Gy X-ray irradiation (Rad Source Technologies RS 2000 Biological Research X-ray Irradiator) on the same day as adoptive transfer of the T cells. Both *in vitro* activated and/or genetically modified antigen-specific CD8^+^ T cells were adoptively transferred 48 hours after initial activation on anti-CD3ε and anti-CD28 (2μg/mL each) coated plates; drug treated T cells were transferred 96 hours after initial activation, with drug or DMSO treatment for the last 72 hours prior to transfer. Drug treated cells were replated at 1×10^6^ cells/mL on fresh anti-CD3ε and anti-CD28 (2μg/mL each) coated plates 48 hours prior to transfer. All transferred cells were washed twice with PBS and resuspended at 20×10^6^ cells/mL in sterile PBS. Irradiated tumor-bearing recipient mice were anesthetized with isoflurane, and 1×10^6^ cells in 50μL PBS were transferred retro-orbitally.

For genetically modified antigen-specific CD8^+^ T cells, initial flow cytometric phenotyping and ratio and knockout confirmation were performed post-transfer. An aliquot of input cells were stained for CD8α, Thy1.1, Thy1.2, CD44, and CD62L to confirm ratios and phenotype transferred cells. Knockout was confirmed after another 48 hours on anti-CD3ε and anti-CD28 (2μg/mL each) coated plates in Complete T Cell Media with 50U/mL IL-2, then staining for the same surface markers as well as PKM1 and PKM2. Flow cytometry data were acquired on a Becton-Dickinson LSRFortessa and analyzed with FlowJo 10 (FlowJo, LLC).

For antigen-specific CD8^+^ T cells treated with drug or DMSO, an aliquot of each of the injected cell populations was stained for CD8α, Thy1.1, Tim3, SlamF6, CD44, CD62L, CCR7, PD-1, CD127, CD8β, Tox, TCF1, Ki67, Eomes, and Tbet to confirm phenotypes of adopted cells, and flow cytometry data acquired on a Becton-Dickinson LSRFortessa and analyzed with FlowJo 10 (FlowJo, LLC).

### Mouse tissue harvest.

At specified time points, mice were euthanized and perfused with PBS. Lungs were then dissected, diced, ground through a 140μm wire mesh (Cell Screen/100mesh, Bellco Glass, Inc.) then filtered through 70μm filters. Lymph nodes were dissected and filtered through 70μm filters. Resultant single-cell suspensions had red blood cells lysed with ACK Lysing Buffer, and cell pellets were washed and resuspended in FACS Buffer. Surface stains, nuclear stains, and stains requiring methanol permeabilization were performed as described above. Intracellular stains for cytokines and effector proteins required stimulation with 1μg/mL Ova_257 – 264_ peptide (InvivoGen) and 2 million freshly isolated C57Bl/6J splenocytes for 5 hours in Complete T Cell Media at 37°C in a humidified incubator, with simultaneous Golgi blocking with Brefeldin A and Monensin. Subsequent staining and acquisition was performed as described above.

### Sorting of mouse lung-infiltrating T cells and TCF1 eGFP reporter-expressing T cells from in vitro co-culture, RNA extraction, and RNA Sequencing analysis.

At specified time points, mice were euthanized and tissue harvested and red blood cells lysed as above. Samples were stained with primary antibodies in the presence of Fc blockers, washed and resuspended in FACS Buffer, and stained with DAPI at 0.2μg/mL. For RNA Sequencing of lung infiltrating T cells in the treatment-naïve HKP1 model, DAPI^−^ TCRβ^+^ CD4^−^ CD8β^+^ cells were sorted into RLT lysis buffer supplemented with b-mercaptoethanol (RNeasy Mini Kit, Qiagen), and frozen at −80°C until RNA extraction using RNeasy Mini Kits with on-column DNA digestion as per the manufacturer’s protocols. For RNA Sequencing of co-adoptively-transferred OT-I^+^ Thy1.1^+/−^ and OT-I^+^ Thy1.1^+/+^ T cells (with and without PKM2 deletion, respectively) in the HKP1-ova-GFP model, DAPI^−^ CD8α^+^ Thy1.1^+^ Thy1.2^+^ and DAPI^−^ CD8α^+^ Thy1.1^+^ Thy1.2^−^ were sorted into Complete T cell Media, washed, resuspended into RLT lysis buffer supplemented with b-mercaptoethanol, and frozen at −80°C until RNA extraction using RNeasy Micro Kits with on-column DNA digestion as per the manufacturer’s protocols.

For RNA Sequencing of cells from the OT-I^+^ Tcf7^GFP+^ strain expressing different levels of a TCF1 eGFP reporter after *in vitro* co-culture with HKP1-ova-GFP tumor cells and treatment with either DMSO, AG1, or 2-DG, cells were harvested, filtered, and washed, and stained as described above, and DAPI^−^ CD8β ^+^ Thy1.2^+^ eGFP + or DAPI^−^ CD8β ^+^ Thy1.2^+^ eGFP− cells were sorted into Complete T cell Media, washed, resuspended into RLT Plus lysis buffer supplemented with b-mercaptoethanol, and frozen at −80°C until RNA extraction using RNeasy Plus Micro Kits with gDNA elimination via gDNA Eliminator spin column as per the manufacturer’s protocols.

For the treatment-naïve dataset, cDNA libraries were generated using the Illumina TruSeq RNA Sample Preparation kit and sequenced single-end 50 bps on the HiSeq2500 sequencer. Tophat2^75^ was used to align raw sequencing reads to the mm9 mouse reference genome. Cufflinks^76,77^ was used to measure transcript abundances in Fragments Per Kilobase of exon model per Million mapped reads (FPKM) with upper-quartile normalization and sequence-specific bias correction. Inter-sample relationships within the CD8 + T cell dataset were evaluated by principal component analysis in R^78^ and visualized using ggplot^79^. Differential gene expression was assessed by utilizing limma^80^, with pairwise comparisons of isolated CD8 + T cells in groups identified by differential tumor burden (Naïve, Day 7, High, and Low). Significance cutoff values were set at absolute log_2_ fold-change ≥ 1, p-value < 0.05, and adjusted p < 0.2. Heatmaps were made using pheatmap and RColorBrewer after normalizing log_2_-transformed FPKM values by the maximum FPKM value for each transcript. Heatmaps in Fig. 1E and Extended Data Fig. 1B used genes from the KEGG Glycolysis/Gluconeogenesis dataset^21^. Mouse Ensembl gene identifiers were mapped to human homologues using biomaRt^81,82^, and Gene Set Enrichment Analysis (GSEA, standard parameters^83^) was performed using Fast Gene Set Enrichment Analysis (fgsea)^84^. Isoform counts were generated with the Partek Flow software (Partek Incorporated) by detecting alternative splicing (Detect alt-splicing) using ANOVA: detecting multiple transcripts in genes, with varying transcript expression based on different groups. Parameters were: Factors-Tumor Burden; Comparisons-High vs Low, High vs Day 7, High vs Naïve, High vs Low + Day 7 + Naïve; Alt-splicing factor-Tumor Burden; Alt-splicing range-2, 100; Filter features by-Lowest average coverage = 1.0; FDR step-up-true; Storey q-value-false; Use only reliable estimation results-Yes; Data has been log transformed with base-None; P-value type-F; Apply normalization-Default (CPM (counts per million); Add 0.0001).

For analysis of the previously published anti-PD-1 treatment dataset^4^ (GSE114300), differential gene expression was performed as previously published, and pairwise comparisons performed between BLI groups 4 and 6 for the Venn diagram found in Fig. 1C using the same significance cutoffs. For GSEA, comparisons were performed between BLI groups 2 and 3, 4 and 6, and 5 and 6 using fgsea^84^.

For the adoptive co-transfer dataset, cDNA libraries were generated using the SMART-Seq v4 Ultra Low Input RNA plus Nextera XT DNA Sample preparation kit and sequenced with paired-end 2×100 cycles on the NovaSeq 6000 sequencer. Raw sequencing reads in BCL format were processed through bcl2fastq 2.19 (Illumina) for FASTQ conversion and demultiplexing. Adaptors were trimmed with cutadapt (version1.18), RNA reads were aligned and mapped to the GRCm38 mouse reference genome by STAR^85^ (version 2.5.2), and transcriptome reconstruction was performed by Cufflinks (version 2.1.1). Raw read counts per gene were extracted using HTSeq-count v0.11.2^85^. Differential gene expression was determined using DESeq2^86^. Principal component analyses were performed using DESeq2. The mouse-specific effect was regressed out using the removeBatchEffect from limma, and PCA was performed on the corrected values. GSEA was performed as above.

For the Tcf7^GFP^ reporter dataset, cDNA libraries were generated using the Illumina Stranded mRNA Prep kit and sequenced with paired-end 2×100 cycles on the NovaSeq 6000 sequencer. Raw sequencing reads in BCL format were processed through bcl2fastq 2.20 (Illumina) for FASTQ conversion and demultiplexing. RNA reads were aligned and mapped to the GRCm39 mouse reference genome by STAR^85^ (version 2.7.10b). The sequencing and mapping quality was evaluated using FastQC (v0.12.1)^87^. Raw read counts per gene were extracted using HTSeq-count (version 2.0.1)^85^. Differential gene expression was determined using a Wald test from DESeq2^86^. Genes with adjusted p-value ≤ 0.05 and fold change ≥ 1.5 were considered significantly differentially expressed. Principal component analyses were performed using DESeq2. Volcano plots were prodused using the R package EnhancedVolcano^88^. GSEA was performed as above.

For identification of upstream regulators, Qiagen Ingenuity Pathway Analysis (IPA, Qiagen, Inc.) software was used. Relevant differential gene expression analyses generated by DESeq2 were uploaded to IPA, then core analysis run using filters of: Species-Mouse; Tissues & Cell Lines-T Lymphocytes; baseMean Cutoff-2000; and pvalue Cutoff-0.1. Upstream Regulators were identified and filtered by absolute Activation z-score > 2.

### LC/MS-based metabolomics.

Steady-state polar metabolite profiling was performed according to a method described in a previous publication^89^. T cells were sorted from co-culture or off CD3ε and anti-CD28 plates as described above, washed twice with ice-cold PBS, then supernatants aspirated and cell pellets frozen at −80°C until analysis. All metabolomics samples were analyzed at the same time. Metabolites were extracted using pre-chilled 80% methanol (−80°C). The extract was dried completely with a Speedvac. The dried sample was redissolved in HPLC grade water before it was applied to the hydrophilic interaction chromatography LC-MS. Metabolites were measured on a Q Exactive Orbitrap mass spectrometer (Thermo Scientific), which was coupled to a Vanquish UPLC system (Thermo Scientific) via an Ion Max ion source with a HESI II probe (Thermo Scientific). A Sequant ZIC-pHILIC column (2.1 mm i.d. × 150 mm, particle size of 5 μm, Millipore Sigma) was used for separation of metabolites. A 2.1 × 20 mm guard column with the same packing material was used for protection of the analytical column. Flow rate was set at 150 μL/min. Buffers consisted of 100% acetonitrile for mobile phase A, and 0.1% NH_4_OH/20 mM CH_3_COONH_4_ in water for mobile phase B. The chromatographic gradient ran from 85–30% A in 20 min followed by a wash with 30% A and re-equilibration at 85% A. The Q Exactive was operated in full scan, polarity-switching mode with the following parameters: the spray voltage 3.0 kV, the heated capillary temperature 300°C, the HESI probe temperature 350°C, the sheath gas flow 40 units, the auxiliary gas flow 15 units. MS data acquisition was performed in the m/z range of 70–1,000, with 70,000 resolution (at 200 m/z). The AGC target was 1e6 and the maximum injection time was 250 ms. The MS data was processed using XCalibur 4.1 (Thermo Scientific) to obtain the metabolite signal intensity for relative quantitation. Metabolites were identified using an in-house library established using chemical standards. Identification required exact mass (within 5ppm) and standard retention times. For downstream analyses, metabolite abundance was normalized by median sample count and day of sample acquisition, with resultant data calculated as fold of NTC-2 abundance.

For glucose isotope labelling experiments, T cells were activated, genetically modified, and co-cultured as described above. At 4 days and 6 days post-initial stimulation, viable CD8 + T cells were sorted from co-culture by FACS as described above into glucose-free RPMI 1640 Medium with L-Glutamine and without Glucose (Gibco) supplemented with 10% heat-inactivated dialyzed (12–14kD) FBS (Atlanta Biologicals), insulin-transferrin-selenium-ethanolamine (ITS-X, Gibco), 1mM sodium pyruvate (Gibco), 100 U/mL penicillin with 100μg/mL streptomycin (Corning), 50μM β-mercaptoethanol (Sigma), and 50U/mL IL-2 (designated Tracer Sort Media). Sorted T cells were then pelleted at 1500rpm for 5 minutes, then resuspended into Tracer Sort Media supplemented with 2000mg/L 1,2–13C2 Glucose (Cambridge Isotope Laboratories), designated Labeling Media, to match the concentration of glucose in Advanced RPMI. 2×10^6^ cells were plated in 1mL of Labeling Media in 1 well of a 12 well plate, and cultured at 37°C for 2 hours^41^. Labelled cells were subsequently transferred to 1.5mL tubes, and pelleted at 6000rpm for 30 seconds then washed with ice cold PBS. Washing was performed once more, then cells pelleted, all liquid removed with a pipette, and cell pellets frozen at −80°C until analysis. Metabolites were extracted using 80% methanol. The supernatants containing polar metabolites were dried down and dissolved in water. Targeted LC/MS analyses were performed on a Q Exactive Orbitrap mass spectrometer (Thermo Scientific) coupled to a Vanquish UPLC system (Thermo Scientific). The Q Exactive operated in polarity-switching mode. A Sequant ZIC-HILIC column (2.1 mm i.d. × 150 mm, Merck) was used for separation of metabolites. Flow rate was set at 150 μL/min. Buffers consisted of 100% acetonitrile for mobile B, and 0.1% NH_4_OH/20 mM CH_3_COONH_4_ in water for mobile A. Gradient ran from 85–30% B in 20 min followed by a wash with 30% B and re-equilibration at 85% B. Data analysis was done using El-MAVEN (v0.12.0). Metabolites and their 13C isotopologues were identified on the basis of exact mass within 5 ppm and standard retention times. Relative metabolite quantitation was performed based on peak area for each metabolite. For downstream analyses, metabolite abundance was normalized per sample by sum of labelled isotope intensities, then calculated as fold of NTC-2 abundance for each day of sample acquisition.

### Metaboanalyst analysis.

Pathway enrichment analyses for carbon tracing experiments were performed using metaboanalyst.ca between April 2023 and July 2023. Labelled isotope counts (m > + 0) for each metabolite were summed and tabulated, then uploaded to metaboanalyst.ca to perform Pathway Analysis on Annotated Features. Samples were normalized by sum, then pathways analyzed using the following parameters: visualization method-scatter plot (testing significant features); enrichment method-global test; topology analysis-relative-betweeness centrality; and reference metabolome-use all compounds in the selected pathway library. The pathway library used was mus musculus (KEGG). According to metaboanalyst.ca, KEGG pathway information was obtained in October 2019.

### Immunocompetent patient-derived tumor organoid (PDTO) analysis.

PDTOs were developed as previously with minor modifications^90^. Fresh tissue samples were washed three times with DMEM and placed in a sterile 3-cm petri dish for mechanical dissection into smaller pieces (2mm diameter) prior to enzymatic digestion. Media containing loose cells or clumps of cells after mechanical dissection were separated from tissue pieces as a “predigest” fraction and used for culture later without enzymatic digestion. Enzymatic digestion was done with collagenase IV media (DMEM (Gibco), 100U/mL penicillin, 100μg/mL streptomycin (Gibco), 250U/mL collagenase IV (Life Technologies), 100μg/mL Primocin (InvivoGen), and 10μmol/L Rock inhibitor Y-27632 (Selleck Chemical Inc.)) in a volume of at least 20 times the tissue volume and incubated on a shaker at 200 rpm at 37°C until the digestion solution turned cloudy, typically 30–45 minutes. The suspension and the predigest fraction were both centrifuged at 1300rpm for 3 minutes. and the cell pellet was washed once with washing media (Advanced DMEM (Gibco), 100U/mL penicillin, 100μg/mL streptomycin (Gibco), 1× Glutamax (Invitrogen), and 1× Hepes (Invitrogen)). The cells in each fraction were resuspended separately in a small volume of tissue-type specific primary culture media (Advanced DMEM (Gibco) with glutamax (1×, Invitrogen), HEPES (Invitrogen), B27 (Gibco), 100U/mL penicillin, 100μg/mL streptomycin (Gibco), 100μg/mL Primocin (InvivoGen), 10% noggin conditioned media, 10% R-spondin conditioned media, 10mM Nicotinamide (Sigma-Aldrich), 1.25mM N-acetylcysteine (Sigma-Aldrich), 1ng/mL Recombinant Human FGF-b (Peprotech), 20ng/mL Recombinant Human FGF-10 (Peprotech), 1μM PGE2 (R&D Systems), 10μM SB202190 (Sigma-Aldrich), 50ng/mL Mouse Recombinant EGF (Invitrogen), 10μM Y-27632 (Selleck Chemicals), 10ng/mL Heregulin Beta-1 (Peprotech), and 500nM A-83–01 (Tocris)). Up to ten 100μL drops of Matrigel/cell suspension were distributed into a 6 well cell suspension culture plate (Gibco). The drops were allowed to polymerize for 30 minutes inside the incubator at 37°C and 5% CO_2_ and afterwards, 3mL tumor type–specific primary culture media were added per well. Fresh culture media was replaced every 3 to 4 days. PDTOs at approximately 300 to 500μm were passaged using TrypLE Express (Gibco) for 10–12 minutes in the water bath at 37°C. Single cells and small cell clusters were replated according to the procedure described above. Monthly mycoplasma screening was performed using the PCR Mycoplasma Detection Kit (Applied Biological Materials). PDTOs were cryopreserved in Recovery Cell Culture Freezing Medium (Gibco) in liquid nitrogen.

### Human T cell rapid expansion protocol (REP).

REP was performed following methods published by Jin and Rosenberg^53^. Briefly, patient peripheral blood mononuclear cell (PBMC) T cells were cultured with allogeneic PBMCs irradiated at 40 or 50Gy at a ratio of 1:200 in a large volume of media (T-75 flask, multiple wells in 6 well plates), supplemented with 3000U/mL recombinant human IL-2 and 1μg/mL anti-CD3. 3μM AG1 or DMSO vehicle control were also included in the media. Half of the media was refreshed every 3 days. After 14 days of culture, cells were harvested, assayed, and cryopreserved as above. An aliquot of these cells was collected and intracellular staining for TCF1 was performed at 48 hours after a dose of drug.

### Human T cell and NSCLC PDTO co-culture.

As previously described in Djskstra et al^52^, tumor reactive T cells were expanded by co-culturing them for 14 days with autologous PDTOs in T cell culture media (RPMI (Gibco), 10% human AB-serum (Gibco), 2mM Glutamax (Gibco), 100 U/mL penicillin, 100 μg/mL streptomycin (Gibco), 25Mm Hepes (Gibco). T cells were challenged with PDTOs at days 0, 7, and 14 of co-culture. Recombinant human IL-2 (300U/ml) was refreshed every 3 days. At day 14, T-cells were rechallenged with PDTOs and functional assays were performed. T cells were either stained to evaluate IFNγ expression or evaluated for killing potential.

### Human PDTO killing assay.

T cells recovered after 2 weeks of co-culture with human NSCLC PDTOs were rechallenged with PDTOs to assess their cytotoxic potential. PDTOs were stained with Cell Trace Far Red (Thermo Fisher Scientific) and seeded in 96 well plates at 5×10^5^ cells/mL in 150μL of medium containing 5μM NucView488 caspase-3 substrate (Biotium). T cells were added at a 5:1 effector:target ratio in 50μL of medium. Plates were imaged using Incucyte (Sartorius) every hour, capturing 4 images per well, over 12 hours. Apoptotic PDTOs were identified as double positive cells for Cell Trace Far Red and NucView488. Baseline spontaneous apoptotic events were subtracted for each experimental condition.

## Statistical analysis.

Results are expressed as mean ± SD. RNA Sequencing gene expression data were analyzed using the limma or DESeq2 packages in R. Gene Set Enrichment Analyses were performed using the fgsea package in R. All statistical tests used are described in the corresponding figure legend. T cells *in vitro* were analyzed by multiple unpaired two-tailed *t*-tests with Holm-Šídák multiple comparisons corrections or two-way ANOVAs with Dunnett’s multiple comparisons correction. *Ex vivo* analyses of adoptively co-transferred T cells were performed using multiple paired two-tailed *t*-tests with Holm-Šídák multiple comparisons corrections or two-way ANOVAs with Tukey’s multiple comparisons correction. Analyses of tumor burden between groups by bioluminescence imaging used two-way ANOVA with Šídák multiple comparisons corrections and Grubbs’ outlier test with alpha = 0.0001. Mouse overall survival was evaluated by Log-rank (Mantel-Cox) test. Metabolomics data were analyzed using multiple unpaired two-tailed *t*-tests. All statistical analyses not performed in R were done using the GraphPad Prism 9 statistical program (GraphPad Software, LLC). P values < 0.05 were considered significant.

## Availability.

Source data have been provided as Source Data files, with all other data supporting the findings available from the corresponding author on reasonable request. R code used in this manuscript is available on reasonable request. All other reagents are available either commercially or from the corresponding author on reasonable request. RNA Sequencing data are available in the Gene Expression Omnibus (http://www.ncbi.nlm.nih.gov/gds). Associated accession numbers are: Non-treated bulk RNA Sequencing: GSE218141; Anti-PD-1 bulk RNA Sequencing: GSE114300 (Reference^4^); Bulk RNA Sequencing of isolated adoptively co-transferred TILs: GSE216675; Bulk RNA Sequencing of TCF1 reporter eGFP + and eGFP-T cells from *in vitro* co-culture and metabolic manipulation: GSE238203.

## Antibodies, Dyes, Drugs, and Recombinant Proteins Used

**Table:** 

Use	Species	Reagent	Clone or Catalogue Number	Company	Dilution/Concentration
Flow Cytometry	Mouse	anti-CD45	30-F11	Biolegend	1:100
Flow Cytometry	Mouse	anti-CD11c	N418	Biolegend	1:100
Flow Cytometry	Mouse	anti-I-A/I-E	M5/114.15.2	Biolegend	1:100
Flow Cytometry	Mouse	anti-TCR β	H57–597	Biolegend	1:100
Flow Cytometry	Mouse	anti-CD3	17A2	Biolegend	1:100
Flow Cytometry	Mouse	anti-CD4	RM4–5	Biolegend	1:100
Flow Cytometry	Mouse	anti-CD8a	53 – 6.7	Biolegend	1:100
Flow Cytometry	Mouse	anti-CD8b	YTS156.7.7	Biolegend	1:100
Flow Cytometry	Mouse	anti-Thy1.1	OX-7	Biolegend	1:100
Flow Cytometry	Mouse	anti-Thy1.2	30-H12	Biolegend	1:100
Flow Cytometry	Mouse	anti-CD44	IM7	Biolegend	1:100
Flow Cytometry	Mouse	anti-CD62L	MEL-14	Biolegend	1:100
Flow Cytometry	Mouse	anti-PD-1	29F.1A12	Biolegend	1:100
Flow Cytometry	Mouse	anti-CD39	Duha59	Biolegend	1:100
Flow Cytometry	Mouse	anti-Ly108 (SLAMF6)	330-AJ	Biolegend	1:100
Flow Cytometry	Mouse	anti-TIM-3	RMT3–23	Biolegend	1:100
Flow Cytometry	Mouse	anti-LAG-3	C9B7W	Biolegend	1:100
Flow Cytometry	Mouse	anti-PD-LI	10F.9G2	Biolegend	1:100
Flow Cytometry	Mouse	anti-mouse H-2K^b^ bound to SIINFEKL	25-D1.16	Biolegend	1:100
Flow Cytometry	Mouse	anti-TNFα	MP6-XT22	Biolegend	1:50
Flow Cytometry	Mouse	anti-IFNγ	XMG1.2	Biolegend	1:50
Flow Cytometry	Mouse	anti-GzmB	GB11	Biolegend	1:50
Flow Cytometry	Mouse	anti-IL-2	JES6–5H4	Biolegend	1:50
Flow Cytometry	Mouse	anti-Tbet	4B10	Biolegend	1:50
Flow Cytometry	Mouse	anti-Eomes	Dan11mag	eBioscience	1:50
Flow Cytometry	Mouse	anti-Ki67	11F6	Biolegend	1:50
Flow Cytometry	Mouse	anti-Tox	REA473	Militenyi Biotec	1:50
Flow Cytometry	Mouse	anti-TCF1/TCF7	C63D9	CST	1:50
Flow Cytometry	Mouse	anti-PKM2	D78A4	CST	1:50
Flow Cytometry	Mouse	anti-PKMI	D30G6	CST	1:50
Flow Cytometry	Mouse	Rabbit mAb IgG	DAIE	CST	1:50
Flow Cytometry	Mouse/Human	anti-Foxo1	W20064D	Biolegend	1:50
Flow Cytometry	Mouse/Human	anti-Bach2	ab243148	abeam	1:100
Flow Cytometry	Mouse	Goat anti-Rat AF647	A21247	Invitrogen	1:500
Flow Cytometry	Human	anti-IFNγ	4S.B3	Biolegend	1:75
Flow Cytometry	Human	anti-CD3	UCHT1	Biolegend	1:150
Flow Cytometry	Human	anti-CD8a	RPA-78	Biolegend	1:150
Flow Cytometry	Human	anti-TCF-7/TCF-1	S33–966	BD Biosciences	1:250
Viability - Flow Cytometry	N/A	Zombie Aqua	423102	Biolegend	1:100
Viability - Flow Cytometry	N/A	DAPI	422801	Biolegend	0.2μg/mL
PDTO Killing Assay	N/A	CellTrace Far Red Proliferation Kit	C34564	Thermo Fisher Scientific	1:1000
PDTO Killing Assay	N/A	NucView 488 Caspase-3 Assay Kit for Live Cells	30029	Biotium	5μM
Western Blotting	Mouse	Rabbit anti-PKMI	D30G6	CST	1:1000 in BSA
Western Blotting	Mouse	Rabbit anti-PKM2	D78A4	CST	1:1000 in BSA
Western Blotting	Mouse	Rabbit anti-PKM1/2	#3186	CST	1:500 in BSA
Western Blotting	Mouse	Rabbit anti-alpha Tubulin	#2144	CST	1:2000 in BSA
Western Blotting	Mouse	Rabbit anti-Histone H3	D1H2	CST	1:5000 in milk
Western Blotting	Rabbit	Peroxidase AffiniPure Goat Anti-Rabbit IgG (H + L)	111–035-003	Jackson Immuno-Research Laboratories	1:2000 in milk
*In vivo* blockade	Rat	anti-Mouse PD-1	RMP1–14	BioXCell	250μg/dose
*In vivo* blockade	Rat	lgG2a	2A3	BioXCell	250μg/dose
*In vitro* culture	Mouse	Biotinylated anti-CD3ε	145–2C11	Biolegend	2μg/mL
*In vitro* culture	Mouse	Biotinylated anti-CD28	37.51	Biolegend	2μg/mL
*In vitro* culture	Mouse	Recombinant IL-2	402-ML-100	R&D	50U/mL
*In vitro* culture	Chicken	OVA 257–264	vac-sin	InvivoGen	1μg/mL
*In vitro* culture	N/A	2-Deoxy-D-glucose	D3179–1G	Sigma-Aldrich	2mM in DMSO
*In vitro* culture	N/A	G6PDi-1	SML2980-5MG	Sigma-Aldrich	10–25μM in DMSO
*In vitro* culture	N/A	6-aminonicotinamide	A68203–1G	Sigma-Aldrich	10μM in DMSO
*In vitro* culture	N/A	Oxythiamine chloride hydrochloride	04000–1G	Sigma-Aldrich	10–100μM in DMSO
*In vitro* culture	N/A	G6PD activator AG1	HY-123962	MedChemExpress LLC	3μM in DMSO
Isotope labeling	N/A	1,2–13C2 D-Glucose	CLM-504–0	Cambridge Isotope Laboratories	2000mg/L
Human Rapid Expansion	Human	Recombinant IL-2	200–02	Peprotech	3000U/mL
Human Rapid Expansion	Human	anti-CD3	OKT3	Biolegend	1μg/mL
Enzymatic Digestion	N/A	Collagenase IV	17104019	Life Technologies	250U/mL
Enzymatic Digestion	N/A	ROCK Inhibitor	Y-27632	Selleck Chemical	10μMol/L
Human PDTO Generation	Human	Recombinant Human FGF-β	100–18B	Peprotech	1ng/mL
Human PDTO Generation	Human	Recombinant Human FGF-10	100–26	Peprotech	20ng/mL
Human PDTO Generation	N/A	PGE2	2296	R&D	1μMol/L
Human PDTO Generation	N/A	SB202190	S7067	Sigma-Aldrich	10μMol/L
Human PDTO Generation	Mouse	Recombinant Mouse EGF	μMG-8045	Gibco	50ng/mL
Human PDTO Generation	N/A	ROCK Inhibitor	Y-27632	Selleck Chemical	10μMol/L
Human PDTO Generation	Human	Recombinant Heregulin Beta-1	100–03	Peprotech	10ng/mL
Human PDTO Generation	N/A	A-83–01	2939	Tocris	500nM
Human PDTO Generation	N/A	Nicotinamide	N0636	Sigma-Aldrich	10mM
Human PDTO Generation	N/A	N-acetylcysteine	A9165–5G	Sigma-Aldrich	1.25mM
Human PDTO Generation		10% Noggin conditioned media from transfected HEK 293T	Produced in House		
Human PDTO Generation		10% R-spondin conditioned media from transfected HEK 293T	Produced in House		
Human PDTO Coculture	Human	Recombinant IL-2	200–02	Peprotech	300U/mL

## Guides, Primers, siRNAs, plasmids, and shRNAs Used

**Table:** 

Category	Species / Use	Target	Sequence or ID	Target Exon	Company	Concentration
siRNA	Mouse/Human	DGCR-8/Pasha	5’- CGGGTGGATCATGACATTCCA- 3’	N/A	IDT	20nmol in 1mL
sgRNA	Mouse	Pkm2 #8	5’-ATTCGAGGAACTCCGCCGCC- 3’	10	IDT	2nmol in 20μL
sgRNA	Mouse	Pkm2 #9	5’- CCACGGCGGCAGCTTCTGTG-3’	10	IDT	2nmol in 20μL
shRNA	Mouse	Pkm.1	5’- TTTACACGAAGGTCGACATCCT- 3’	11	Transomic	20μg/1mL reaction
shRNA	Mouse	Pkm.2	5’- TAAGATGCAAACACCATGTCCA- 3’	7	Transomic	20μg/1mL reaction
shRNA	Mouse	Pkm.3	5’- TTAATCATCTCCTTCAGCATCT- 3’	4	Transomic	20μg/1mL reaction
plasmid	Mammal Expression	pCL-Eco	Plasmid #12371	N/A	Addgene	10μg/1mL reaction

## References

[1] Pardoll D. M. The blockade of immune checkpoints in cancer immunotherapy. Nat Rev Cancer 12, 252–264 (2012). 10.1038/nrc3239 22437870 PMC4856023

[2] Sharma P. & Allison J. P. Dissecting the mechanisms of immune checkpoint therapy. Nat Rev Immunol 20, 75–76 (2020). 10.1038/s41577-020-0275-8 31925406

[3] Ribas A. & Wolchok J. D. Cancer immunotherapy using checkpoint blockade. Science 359, 1350–1355 (2018). 10.1126/science.aar4060 29567705 PMC7391259

[4] Markowitz G. J. Immune reprogramming via PD-1 inhibition enhances early-stage lung cancer survival. JCI Insight 3 (2018). 10.1172/jci.insight.96836 PMC6101707 29997286

[5] Philip M. & Schietinger A. CD8(+) T cell differentiation and dysfunction in cancer. Nat Rev Immunol 22, 209–223 (2022). 10.1038/s41577-021-00574-3 34253904 PMC9792152

[6] Blank C. U. Defining ‘T cell exhaustion’. Nat Rev Immunol 19, 665–674 (2019). 10.1038/s41577-019-0221-9 31570879 PMC7286441

[7] Siddiqui I. Intratumoral Tcf1(+)PD-1(+)CD8(+) T Cells with Stem-like Properties Promote Tumor Control in Response to Vaccination and Checkpoint Blockade Immunotherapy. Immunity 50, 195–211 e110 (2019). 10.1016/j.immuni.2018.12.021 30635237

[8] Miller B. C. Subsets of exhausted CD8(+) T cells differentially mediate tumor control and respond to checkpoint blockade. Nat Immunol 20, 326–336 (2019). 10.1038/s41590-019-0312-6 30778252 PMC6673650

[9] van Loosdregt J. & Coffer P. J. The Role of WNT Signaling in Mature T Cells: T Cell Factor Is Coming Home. J Immunol 201, 2193–2200 (2018). 10.4049/jimmunol.1800633 30301837

[10] Gattinoni L. Wnt signaling arrests effector T cell differentiation and generates CD8 + memory stem cells. Nat Med 15, 808–813 (2009). 10.1038/nm.1982 19525962 PMC2707501

[11] Escobar G., Mangani D. & Anderson A. C. T cell factor 1: A master regulator of the T cell response in disease. Sci Immunol 5 (2020). 10.1126/sciimmunol.abb9726 PMC8221367 33158974

[12] Kurtulus S. Checkpoint Blockade Immunotherapy Induces Dynamic Changes in PD-1(−)CD8(+) Tumor-Infiltrating T Cells. Immunity 50, 181–194 e186 (2019). 10.1016/j.immuni.2018.11.014 30635236 PMC6336113

[13] Kishton R. J., Sukumar M. & Restifo N. P. Metabolic Regulation of T Cell Longevity and Function in Tumor Immunotherapy. Cell Metab 26, 94–109 (2017). 10.1016/j.cmet.2017.06.016 28683298 PMC5543711

[14] Reina-Campos M., Scharping N. E. & Goldrath A. W. CD8(+) T cell metabolism in infection and cancer. Nat Rev Immunol 21, 718–738 (2021). 10.1038/s41577-021-00537-8 33981085 PMC8806153

[15] van der Windt G. J. & Pearce E. L. Metabolic switching and fuel choice during T-cell differentiation and memory development. Immunol Rev 249, 27–42 (2012). 10.1111/j.1600-065X.2012.01150.x 22889213 PMC3645891

[16] Wang R. & Green D. R. Metabolic reprogramming and metabolic dependency in T cells. Immunol Rev 249, 14–26 (2012). 10.1111/j.1600-065X.2012.01155.x 22889212 PMC3422760

[17] Hermans D. Lactate dehydrogenase inhibition synergizes with IL-21 to promote CD8(+) T cell stemness and antitumor immunity. Proc Natl Acad Sci U S A 117, 6047–6055 (2020). 10.1073/pnas.1920413117 32123114 PMC7084161

[18] Pearce E. L. Enhancing CD8 T-cell memory by modulating fatty acid metabolism. Nature 460, 103–107 (2009). 10.1038/nature08097 19494812 PMC2803086

[19] Sukumar M. Inhibiting glycolytic metabolism enhances CD8 + T cell memory and antitumor function. J Clin Invest 123, 4479–4488 (2013). 10.1172/JCI69589 24091329 PMC3784544

[20] Choi H. Transcriptome analysis of individual stromal cell populations identifies stroma-tumor crosstalk in mouse lung cancer model. Cell Rep 10, 1187–1201 (2015). 10.1016/j.celrep.2015.01.040 25704820

[21] Kanehisa M. & Goto S. KEGG: kyoto encyclopedia of genes and genomes. Nucleic Acids Res 28, 27–30 (2000). 10.1093/nar/28.1.27 10592173 PMC102409

[22] Vardhana S. A. Impaired mitochondrial oxidative phosphorylation limits the self-renewal of T cells exposed to persistent antigen. Nat Immunol 21, 1022–1033 (2020). 10.1038/s41590-020-0725-2 32661364 PMC7442749

[23] Horton B. L. Lack of CD8(+) T cell effector differentiation during priming mediates checkpoint blockade resistance in non-small cell lung cancer. Sci Immunol 6, eabi8800 (2021). 10.1126/sciimmunol.abi8800 34714687 PMC10786005

[24] DuPage M. Endogenous T cell responses to antigens expressed in lung adenocarcinomas delay malignant tumor progression. Cancer Cell 19, 72–85 (2011). 10.1016/j.ccr.2010.11.011 21251614 PMC3069809

[25] Hogquist K. A. T cell receptor antagonist peptides induce positive selection. Cell 76, 17–27 (1994). 10.1016/0092-8674(94)90169-4 8287475

[26] Jurica M. S. The allosteric regulation of pyruvate kinase by fructose-1,6-bisphosphate. Structure 6, 195–210 (1998). 10.1016/s0969-2126(98)00021-5 9519410

[27] Ban Y. Radiation-activated secretory proteins of Scgb1a1 (+) club cells increase the efficacy of immune checkpoint blockade in lung cancer. Nat Cancer 2, 919–931 (2021). 10.1038/s43018-021-00245-1 34917944 PMC8670735

[28] Luckey C. J. Memory T and memory B cells share a transcriptional program of self-renewal with long-term hematopoietic stem cells. Proc Natl Acad Sci U S A 103, 3304–3309 (2006). 10.1073/pnas.0511137103 16492737 PMC1413911

[29] Liberzon A. The Molecular Signatures Database (MSigDB) hallmark gene set collection. Cell Syst 1, 417–425 (2015). 10.1016/j.cels.2015.12.004 26771021 PMC4707969

[30] Ecker C. Differential Reliance on Lipid Metabolism as a Salvage Pathway Underlies Functional Differences of T Cell Subsets in Poor Nutrient Environments. Cell Rep 23, 741–755 (2018). 10.1016/j.celrep.2018.03.084 29669281 PMC5929999

[31] Pucino V. Lactate Buildup at the Site of Chronic Inflammation Promotes Disease by Inducing CD4(+) T Cell Metabolic Rewiring. Cell Metab 30, 1055–1074 e1058 (2019). 10.1016/j.cmet.2019.10.004 31708446 PMC6899510

[32] Seki S. M. Modulation of PKM activity affects the differentiation of T(H)17 cells. Sci Signal 13 (2020). 10.1126/scisignal.aay9217 PMC8040370 33109748

[33] Kono M. Pyruvate kinase M2 is requisite for Th1 and Th17 differentiation. JCI Insight 4 (2019). 10.1172/jci.insight.127395 PMC6629104 31217348

[34] Angiari S. Pharmacological Activation of Pyruvate Kinase M2 Inhibits CD4(+) T Cell Pathogenicity and Suppresses Autoimmunity. Cell Metab 31, 391–405 e398 (2020). 10.1016/j.cmet.2019.10.015 31761564 PMC7001035

[35] Stincone A. The return of metabolism: biochemistry and physiology of the pentose phosphate pathway. Biol Rev Camb Philos Soc 90, 927–963 (2015). 10.1111/brv.12140 25243985 PMC4470864

[36] Bouzier-Sore A. K. & Bolanos J. P. Uncertainties in pentose-phosphate pathway flux assessment underestimate its contribution to neuronal glucose consumption: relevance for neurodegeneration and aging. Front Aging Neurosci 7, 89 (2015). 10.3389/fnagi.2015.00089 26042035 PMC4436897

[37] Jang C., Chen L. & Rabinowitz J. D. Metabolomics and Isotope Tracing. Cell 173, 822–837 (2018). 10.1016/j.cell.2018.03.055 29727671 PMC6034115

[38] Xia J., Psychogios N., Young N. & Wishart D. S. MetaboAnalyst: a web server for metabolomic data analysis and interpretation. Nucleic Acids Res 37, W652–660 (2009). 10.1093/nar/gkp356 19429898 PMC2703878

[39] Pang Z. MetaboAnalyst 5.0: narrowing the gap between raw spectra and functional insights. Nucleic Acids Res 49, W388–W396 (2021). 10.1093/nar/gkab382 34019663 PMC8265181

[40] Daneshmandi S. Blockade of 6-phosphogluconate dehydrogenase generates CD8(+) effector T cells with enhanced anti-tumor function. Cell Rep 34, 108831 (2021). 10.1016/j.celrep.2021.108831 33691103 PMC8051863

[41] Ghergurovich J. M. A small molecule G6PD inhibitor reveals immune dependence on pentose phosphate pathway. Nat Chem Biol 16, 731–739 (2020). 10.1038/s41589-020-0533-x 32393898 PMC7311271

[42] Lu C. G6PD functions as a metabolic checkpoint to regulate granzyme B expression in tumor-specific cytotoxic T lymphocytes. J Immunother Cancer 10 (2022). 10.1136/jitc-2021-003543 PMC8753452 35017152

[43] Wang R. The transcription factor Myc controls metabolic reprogramming upon T lymphocyte activation. Immunity 35, 871–882 (2011). 10.1016/j.immuni.2011.09.021 22195744 PMC3248798

[44] Hwang S. Correcting glucose-6-phosphate dehydrogenase deficiency with a small-molecule activator. Nat Commun 9, 4045 (2018). 10.1038/s41467-018-06447-z 30279493 PMC6168459

[45] Yang Q. TCF-1 upregulation identifies early innate lymphoid progenitors in the bone marrow. Nat Immunol 16, 1044–1050 (2015). 10.1038/ni.3248 26280998 PMC4575643

[46] Yao C. BACH2 enforces the transcriptional and epigenetic programs of stem-like CD8(+) T cells. Nat Immunol 22, 370–380 (2021). 10.1038/s41590-021-00868-7 33574619 PMC7906956

[47] Roychoudhuri R. BACH2 regulates CD8(+) T cell differentiation by controlling access of AP-1 factors to enhancers. Nat Immunol 17, 851–860 (2016). 10.1038/ni.3441 27158840 PMC4918801

[48] Rao R. R., Li Q., Gubbels Bupp M. R. & Shrikant P. A. Transcription factor Foxo1 represses T-bet-mediated effector functions and promotes memory CD8(+) T cell differentiation. Immunity 36, 374–387 (2012). 10.1016/j.immuni.2012.01.015 22425248 PMC3314246

[49] Kim M. V., Ouyang W., Liao W., Zhang M. Q. & Li M. O. The transcription factor Foxo1 controls central-memory CD8 + T cell responses to infection. Immunity 39, 286–297 (2013). 10.1016/j.immuni.2013.07.013 23932570 PMC3809840

[50] Delpoux A., Lai C. Y., Hedrick S. M. & Doedens A. L. FOXO1 opposition of CD8(+) T cell effector programming confers early memory properties and phenotypic diversity. Proc Natl Acad Sci U S A 114, E8865–E8874 (2017). 10.1073/pnas.1618916114 28973925 PMC5651728

[51] Podaza E. Novel co-culture strategies of tumor organoids with autologous T-cells reveal clinically relevant combinations of immune-checkpoint and targeted therapies. bioRxiv, 2023.2007.2005.546622 (2023). 10.1101/2023.07.05.546622

[52] Dijkstra K. K. Generation of Tumor-Reactive T Cells by Co-culture of Peripheral Blood Lymphocytes and Tumor Organoids. Cell 174, 1586–1598 e1512 (2018). 10.1016/j.cell.2018.07.009 30100188 PMC6558289

[53] Jin J. Simplified method of the growth of human tumor infiltrating lymphocytes in gas-permeable flasks to numbers needed for patient treatment. J Immunother 35, 283–292 (2012). 10.1097/CJI.0b013e31824e801f 22421946 PMC3315105

[54] Toriyama K. T cell-specific deletion of Pgam1 reveals a critical role for glycolysis in T cell responses. Commun Biol 3, 394 (2020). 10.1038/s42003-020-01122-w 32709928 PMC7382475

[55] Siska P. J. Suppression of Glut1 and Glucose Metabolism by Decreased Akt/mTORC1 Signaling Drives T Cell Impairment in B Cell Leukemia. J Immunol 197, 2532–2540 (2016). 10.4049/jimmunol.1502464 27511728 PMC5010978

[56] Ho P. C. Phosphoenolpyruvate Is a Metabolic Checkpoint of Anti-tumor T Cell Responses. Cell 162, 1217–1228 (2015). 10.1016/j.cell.2015.08.012 26321681 PMC4567953

[57] Gemta L. F. Impaired enolase 1 glycolytic activity restrains effector functions of tumor-infiltrating CD8(+) T cells. Sci Immunol 4 (2019). 10.1126/sciimmunol.aap9520 PMC6824424 30683669

[58] Chang C. H. Posttranscriptional control of T cell effector function by aerobic glycolysis. Cell 153, 1239–1251 (2013). 10.1016/j.cell.2013.05.016 23746840 PMC3804311

[59] Telang S. Small molecule inhibition of 6-phosphofructo-2-kinase suppresses t cell activation. J Transl Med 10, 95 (2012). 10.1186/1479-5876-10-95 22591674 PMC3441391

[60] Quinn W. J. 3rd Lactate Limits T Cell Proliferation via the NAD(H) Redox State. Cell Rep 33, 108500 (2020). 10.1016/j.celrep.2020.108500 33326785 PMC7830708

[61] Mehta M. M. Hexokinase 2 is dispensable for T cell-dependent immunity. Cancer Metab 6, 10 (2018). 10.1186/s40170-018-0184-5 30140438 PMC6098591

[62] Gu M. NF-kappaB-inducing kinase maintains T cell metabolic fitness in antitumor immunity. Nat Immunol 22, 193–204 (2021). 10.1038/s41590-020-00829-6 33398181 PMC7855506

[63] Lu S. PKM2-dependent metabolic reprogramming in CD4(+) T cells is crucial for hyperhomocysteinemia-accelerated atherosclerosis. J Mol Med (Berl) 96, 585–600 (2018). 10.1007/s00109-018-1645-6 29732501

[64] Utzschneider D. T. T Cell Factor 1-Expressing Memory-like CD8(+) T Cells Sustain the Immune Response to Chronic Viral Infections. Immunity 45, 415–427 (2016). 10.1016/j.immuni.2016.07.021 27533016

[65] Shan Q. Tcf1 preprograms the mobilization of glycolysis in central memory CD8(+) T cells during recall responses. Nat Immunol 23, 386–398 (2022). 10.1038/s41590-022-01131-3 35190717 PMC8904300

[66] Im S. J. Defining CD8 + T cells that provide the proliferative burst after PD-1 therapy. Nature 537, 417–421 (2016). 10.1038/nature19330 27501248 PMC5297183

[67] Li W. NADPH levels affect cellular epigenetic state by inhibiting HDAC3-Ncor complex. Nat Metab 3, 75–89 (2021). 10.1038/s42255-020-00330-2 33462516

[68] Tay R. E. Hdac3 is an epigenetic inhibitor of the cytotoxicity program in CD8 T cells. J Exp Med 217 (2020). 10.1084/jem.20191453 PMC7336313 32374402

[69] Gray S. M., Amezquita R. A., Guan T., Kleinstein S. H. & Kaech S. M. Polycomb Repressive Complex 2-Mediated Chromatin Repression Guides Effector CD8(+) T Cell Terminal Differentiation and Loss of Multipotency. Immunity 46, 596–608 (2017). 10.1016/j.immuni.2017.03.012 28410989 PMC5457165

[70] Pace L. The epigenetic control of stemness in CD8(+) T cell fate commitment. Science 359, 177–186 (2018). 10.1126/science.aah6499 29326266

[71] Kahan S. M. Intrinsic IL-2 production by effector CD8 T cells affects IL-2 signaling and promotes fate decisions, stemness, and protection. Sci Immunol 7, eabl6322 (2022). 10.1126/sciimmunol.abl6322 35148200 PMC8923238

[72] Chang K., Marran K., Valentine A. & Hannon G. J. Packaging shRNA retroviruses. Cold Spring Harb Protoc 2013, 734–737 (2013). 10.1101/pdb.prot076448 23906912

[73] Jacobi A. M. Simplified CRISPR tools for efficient genome editing and streamlined protocols for their delivery into mammalian cells and mouse zygotes. Methods 121–122, 16–28 (2017). 10.1016/j.ymeth.2017.03.021 PMC5761324 28351759

[74] Kloss C. C. Dominant-Negative TGF-beta Receptor Enhances PSMA-Targeted Human CAR T Cell Proliferation And Augments Prostate Cancer Eradication. Mol Ther 26, 1855–1866 (2018). 10.1016/j.ymthe.2018.05.003 29807781 PMC6037129

[75] Kim D. TopHat2: accurate alignment of transcriptomes in the presence of insertions, deletions and gene fusions. Genome Biol 14, R36 (2013). 10.1186/gb-2013-14-4-r36 23618408 PMC4053844

[76] Trapnell C. Differential analysis of gene regulation at transcript resolution with RNA-seq. Nat Biotechnol 31, 46–53 (2013). 10.1038/nbt.2450 23222703 PMC3869392

[77] Trapnell C. Transcript assembly and quantification by RNA-Seq reveals unannotated transcripts and isoform switching during cell differentiation. Nat Biotechnol 28, 511–515 (2010). 10.1038/nbt.1621 20436464 PMC3146043

[78] Team R. C. R: A Language and environment for statistical computing., (R Foundation for Statistical Computing, 2022).

[79] Wickham H. ggplot2: Elegant Graphics for Data Analysis. (Springer-Verlag New York, 2016).

[80] Ritchie M. E. limma powers differential expression analyses for RNA-sequencing and microarray studies. Nucleic Acids Res 43, e47 (2015). 10.1093/nar/gkv007 25605792 PMC4402510

[81] Durinck S., Spellman P. T., Birney E. & Huber W. Mapping identifiers for the integration of genomic datasets with the R/Bioconductor package biomaRt. Nat Protoc 4, 1184–1191 (2009). 10.1038/nprot.2009.97 19617889 PMC3159387

[82] Durinck S. BioMart and Bioconductor: a powerful link between biological databases and microarray data analysis. Bioinformatics 21, 3439–3440 (2005). 10.1093/bioinformatics/bti525 16082012

[83] Mootha V. K. PGC-1alpha-responsive genes involved in oxidative phosphorylation are coordinately downregulated in human diabetes. Nat Genet 34, 267–273 (2003). 10.1038/ng1180 12808457

[84] Korotkevic G., Sukhov V. & Sergushichev A. Fast gene set enrichment analysis. bioRxiv (2019). 10.1101/060012

[85] Anders S., Pyl P. T. & Huber W. HTSeq–a Python framework to work with high-throughput sequencing data. Bioinformatics 31, 166–169 (2015). 10.1093/bioinformatics/btu638 25260700 PMC4287950

[86] Love M. I., Huber W. & Anders S. Moderated estimation of fold change and dispersion for RNA-seq data with DESeq2. Genome Biol 15, 550 (2014). 10.1186/s13059-014-0550-8 25516281 PMC4302049

[87] Andrews S. FastQC: A Quality Control Tool for High Throughput Sequence Data [Online], <http://www.bioinformatics.babraham.ac.uk/projects/fastqc/> (2010).

[88] Blighe K., Rana S. & Lewis M. EnhancedVolcano: Publication-ready volcano plots with enhanced colouring and labeling., <https://github.com/kevinblighe/EnhancedVolcano> (2018).

[89] Chen W. W., Freinkman E., Wang T., Birsoy K. & Sabatini D. M. Absolute Quantification of Matrix Metabolites Reveals the Dynamics of Mitochondrial Metabolism. Cell 166, 1324–1337 e1311 (2016). 10.1016/j.cell.2016.07.040 27565352 PMC5030821

[90] Pauli C. Personalized In vitro and In vivo Cancer Models to Guide Precision Medicine. Cancer Discov 7, 462–477 (2017). 10.1158/2159-8290.CD-16-1154 28331002 PMC5413423

